# Long-term seasonal inhomogeneity and recent patterns in air-sea conditions and tropical cyclone activities over the Bay of Bengal

**DOI:** 10.1038/s41598-026-52751-w

**Published:** 2026-05-12

**Authors:** Bijan Kumar Das, Moumita Dinda, Anushri Pal

**Affiliations:** 1 Department of Mathematics, Midnapore College (Autonomous), Midnapore, 721101 India; 2 Department of Earth and Atmospheric Sciences, National Institute Technology Rourkela, Rourkela, 769008 India

**Keywords:** Air-sea interactions, Upper-ocean thermodynamics, Tropical cyclone, Seasonal inhomogeneity, Recent patterns, Bay of Bengal, Climate sciences, Environmental sciences, Ocean sciences

## Abstract

The Bay of Bengal (BoB) covers about 0.7% of the world ocean area but produces almost 4% of the Tropical Cyclones (TCs) of the world. Moreover, about 75% TCs of the North Indian Ocean (NIO) generate in the BoB. The TCs in the BoB have pronounced seasonal variation and predominantly occur in two seasons, Pre-Indian Summer Monsoon (ISM, ~ 23%) and post-ISM (~ 65%). This study presents a quantitative analysis to discuss the seasonality in ocean, atmospheric conditions and the TC activities over the BoB using long-term (1982–2022) data. The quantitative statistical analysis also highlights the changing patterns of the air-sea and TC parameters in recent years (2001–2022). The results show that the air-sea conditions have a strong seasonal variation during pre- and post-ISM over the analysis period. In recent years, the air-sea conditions followed a strong changing pattern which leads the major shift in TC frequency, movements and intensifications. Overall, the study shows that the seasonal inhomogeneity in the upper ocean thermodynamics and atmospheric conditions trigger the seasonal variations in TC activity over the BoB. The upsurges in intensified TC activities over the BoB in the recent years is also reflected in the air-sea forcing conditions.

**Supplementary Information:**

The online version contains supplementary material available at 10.1038/s41598-026-52751-w.

## Introduction

The Bay of Bengal (BoB) is the eastern basin of the North Indian Ocean (NIO). In NIO, the western (i.e. Arabian Sea) and eastern (i.e. BoB) basins generally show contrasting seawater properties^1,2^. The NIO produces almost 6% of the Tropical Cyclones (TCs) of the world annually but faces 75% of total fatalities^3^. The BoB, which is considered one of the large TC producing basins in the world, annually contributes 75% TCs and 80% fatalities occur over the NIO. Therefore, better understanding of the activities, properties, patterns and forcings of the TCs over the BoB is always helpful for livelihood and blue economy of the neighbouring countries like Sri Lanka, India, Bangladesh and Myanmar^4^.

The cyclones in the BoB follows a strong seasonal variation and the maximum number of TCs occur either in pre-Indian Summer Monsoon (pre-ISM, March-April-May) or in post-ISM (October-November-December) period^5^. For example, to quantify, this study has shown the occurrence of 23.3% and 64.6% TCs in pre- and post-ISM, respectively in last 41 years (1982–2022), while the rest of 12.1% TCs occurred during ISM (June-July-August-September) and the months of January-February. Earlier studies have reported that the overall TC frequency over the NIO as well as BoB has decreased but the rapid intensifications have been increased since the year 2000^3,5–8^. Again, the pre-ISM TCs over the BoB are found to be stronger and more intensified compared to the post-ISM^9^. The studies have mentioned that the movement of intense TCs over BoB are shifted from north and northeast to northwest ward during pre-ISM, while the TC movements are generally northwest ward in post-ISM^10,11^. The Indian states of Andhra Pradesh (AP) and Odissa (OD) coastlines remains the most vulnerable for the landfall of rapid intensified TCs of the BoB^7,12,13^.

A cyclone is primarily an atmospheric event but it gets the energy supply from the upper layers of the ocean (mostly 26 °C isothermal layer) during its genesis, intensification and movement^14^. Thus, favourable upper ocean conditions, strong energy and moisture supply to the atmosphere are fundamental forcings from the ocean to influence the TC genesis and activities. Previous studies have shown that the ocean state conditions such as warm core eddies, upper ocean temperature, Ocean Heat Content (OHC) and Cyclone Heat Potential (CHP) in the BoB influence the pre-ISM TCs the most^5,9,15,16^. On the contrary, post-ISM cyclones in the BoB are more affected by atmospheric convective heat flux, limited Sea Surface Temperature (SST) cooling and thick barrier layer^16,17^. Studies have reported that 10–30 m thick barrier layer and ~ 6–8 × 10^5^ kJ/m^2^ CHP can favour TC intensifications^18^. Predicting upper ocean properties of the BoB remains challenging due to its freshwater driven mixing and stratification^19,20^. The BoB has experienced ~ 0.5°-0.6 °C SST increase in last four decades^21,22^. But the strong salinity stratification sometimes prevents convective thermal mixing even when the surface is cooler than the subsurface resulting a subsurface heat trap and modification in air-sea heat exchange^20^. Additionally, the temperature and salinity distribution over the BoB plays a major role to determine the upper layer conditions and their spatiotemporal variation which mostly influence the dynamics of air-sea interactions in the BoB^22^. So, better prediction of the TCs over the BoB also requires better understanding of the upper ocean thermodynamics of the BoB.

As atmospheric event, the tropical cyclogenesis and its sustainability require favourable atmospheric conditions like presence of strong energy fluxes, moisture content and vertical wind shear^9,11,16,23^. Outgoing heat fluxes namely Long Wave Radiation Flux (LWRF), Latent Heat Flux (LHF) and Sensible Heat Flux (SHF) have crucial role to play to supply energy and moisture from ocean to the atmosphere. The relation between the OHC over the 26° isotherm and LWRF suggests that it is the atmospheric forces which initiates the changes in OHC and in some cases during fall and winter seasons, OHC leads atmospheric convections^24^. Again, the high outward gradient (from cyclone core) in radiative flux convergence ($\:\ge\:$60 W/m^2^) can intensifies the TCs^25^. The low vertical wind shear is found much favourable for pre-ISM TC genesis and intensification^9^. On the climatic events, previous studies have argued of having more (less) pronounced TC activities during La Niña (El Niño) years, while the cyclogenesis points have shifted to east (west) of 87°E latitude^13^. The atmospheric conditions, which influence the TC activities, are predominately the results of air-sea interactions and winds. Better understanding of the seasonal variability in these atmospheric parameters in association with the upper ocean conditions may lead to a comprehensive understanding of the air-sea conditions for better predicting the TC activities.

The cyclone related studies over the BoB (as well as over NIO) have drawn more attentions from the researcher community since the last decade. Currently, various studies are available which have discussed either particular TC events or the TC activities with possible ocean/ atmospheric influences over the BoB. The available studies have undoubtably advanced the understanding of the seasonal TC activities over the region. Still long-term quantitative studies are not available over the BoB to comprehensively discuss the seasonal (pre- and post-ISM) TC activities along with the air-sea condition analysis.

In particular, this study aims to present a long-term (1982–2022) quantitative approach to analyze the seasonality (pre- and post-ISM) of the TC activities and associated ocean-atmospheric forcing conditions. The study also highlights the changing patterns of both the air-sea conditions as well as TC activities quantitively in recent years (2001–2022). The objectives are important for better understanding of the upper ocean conditions, associated air-sea interactions and resulted TC activities, which are fundamental to the improved prediction of the TC genesis and intensification, and also support the United Nation’s Ocean Decade for Sustainable Development goal of ‘*A predicted ocean*’.

Therefore, firstly, the study analysed the upper ocean thermodynamic parameters of the BoB and their seasonality during pre- and post-ISM of 1982–2022 that influence the air-sea interactions and hence TC activities over the region. The seasonal variations of the atmospheric parameters, which are affected by upper ocean thermodynamics and directly contributes to the TC activities, are presented thereafter. Then the TC activities and its intensifications are discussed in the context of the seasonal air-sea influences. And finally, the study highlights the patterns in the oceanic response to the atmosphere and the TC activities in the recent period (2001–2022) compared to the previous period (1982–2000). In this study, the year 2000 is considered as a change point (year) and quantified in the recent pattern analysis based on the earlier studies of TC activities over the region^3,7,8^. Although the earlier period consists of 19 years while the recent period has 22 years, the statistical comparison intends to represent the overall differences in the patterns and changing characteristics of the parameters between these two periods.

The findings of this study and associated discussions are presented in the *Results* and the *Discussions* sections, respectively. The *Conclusions* and *Methods* are giver thereafter. The details of the datasets used in the analysis are mentioned in the *Data availability statement*. The statistical inferences used in the *Results* and *Discussions* sections are provided in the Tables S1-S6 of the *Supplementary Information*.

## Results

### Upper ocean thermodynamic conditions

#### Temperature

**Fig. 1:**
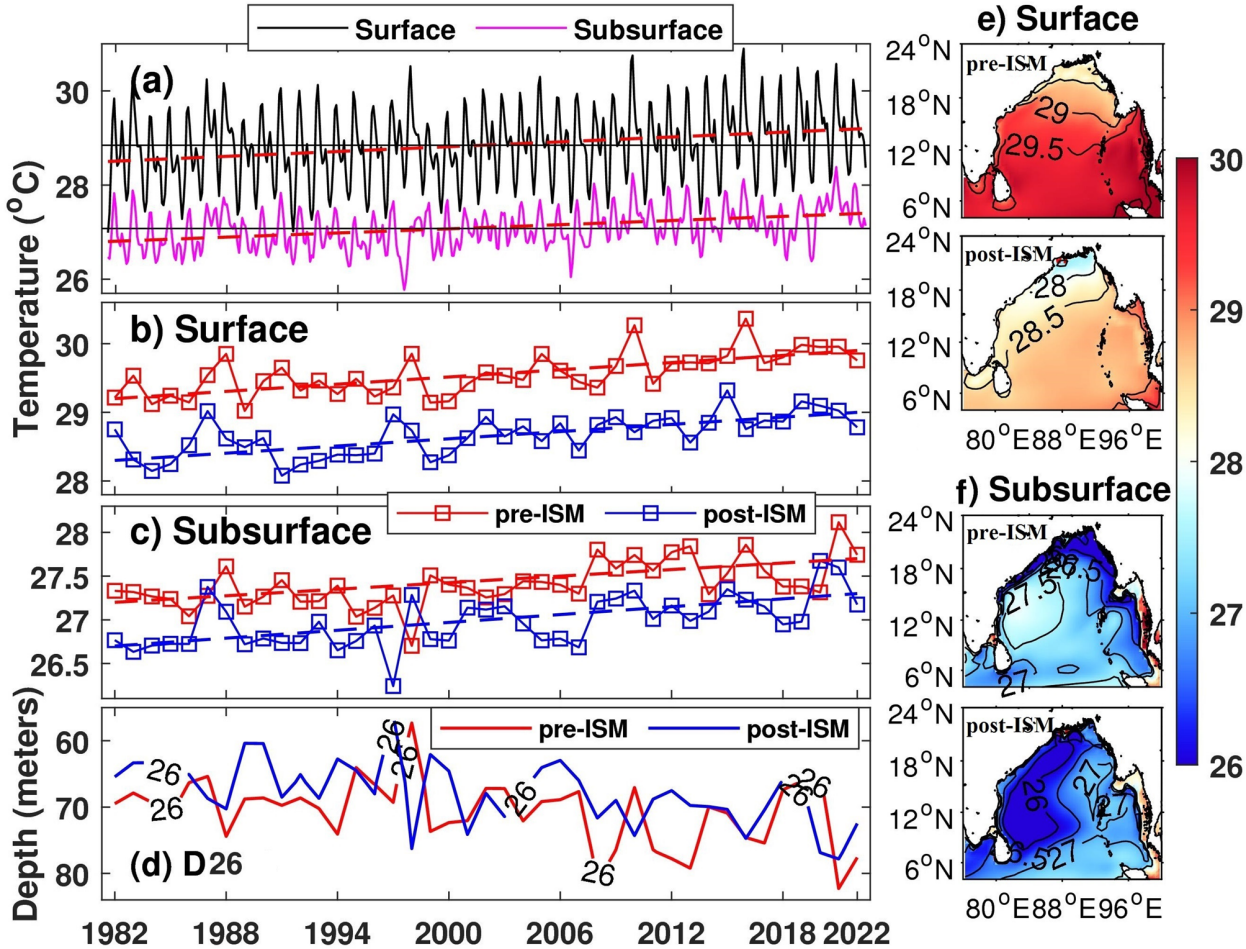
(**a**) Monthly mean surface (black) and subsurface (depth averaged of upper 100 m, magenta) temperature (in °C) time series over the BoB. Red colour dotted lines indicate the linear trend of data and black horizontal lines are data mean (28.84 °C for surface and 27.08 °C for subsurface). (**b**,**c**) Seasonal mean surface (in b) and subsurface (in c) temperature during pre- (red) and post-ISM (blue). (**d**) The region averaged 26 °C isothermal layer depth (D26) during pre- and post-ISM. (**e**,** f**) The seasonal climatology of spatial SST (in e) and SubST (in f) patterns over the BoB during pre- and post-ISM.

Earlier studies have reported that the SST over the BoB has been increased approximately 0.5°- 0.6 °C in the last four to five decades^21,22^. The increase in surface temperature can substantially modulate the subsurface temperature, OHC, CHP and the air-sea interactions, which potentially impact the tropical cyclogenesis and cyclone intensifications^3,5,16^. Here, the surface and subsurface (depth averaged in 0–100 m and referred as SubST) temperature analysis over the BoB during 1982–2022 is shown in Fig. 1 (also see Tables S1, S2 in the Supplementary Information for quantitative annual and seasonal statistical values). The monthly SST shows a mean value of 28.84 °C during 1982–2022 and it has increased with a slope of 0.016 °C/year (p-value $\:6.79\times\:{10}^{-9}$, Fig. 1a, Table S1). In case of SubST, the mean value remained 27.08 °C with an increasing slope of 0.013 °C/year (p-value $\:5.97\times\:{10}^{-18}$). This rising trend caused an increase of ~ 0.66 °C surface temperature over the analysis period, while the subsurface temperature increment is 0.54 °C.

The seasonal mean SST during pre- and post-ISM (Fig. 1b) have increased almost in a similar rate (slopes are 0.017 °C/year with p-value $\:3.25\times\:{10}^{-6}$ and 0.016 °C/year with p-value $\:5.52\times\:{10}^{-6}$, respectively) during 1982-2022 (Table S2). In spite of having ~ 1 °C temperature difference (mean), both pre- and post-ISM SST has increased ~ 0.7 °C. On the contrary, the post-ISM SubST has seen increasing in a faster rate (slope is 0.014 °C/ year with p-value $\:6.04\times\:{10}^{-5}$) than the pre-ISM (slope is 0.012 °C/year with p-value $\:9.66\times\:{10}^{-5}$, Fig. 1c). As a results, the SubST in post-ISM has increased 0.6 °C in these 41 years (1982-2022) compared to 0.5 °C in pre-ISM. The surface temperature patterns over the BoB in Fig. 1e show a similar spatial distribution of temperature during pre- and post-ISM with a temperature difference of ~ 1–1.5 °C. On the other side, the spatial distribution of subsurface temperature in both the seasons is mostly concentrated in the northwestern BoB, where the pre-ISM is ~ 2 °C warmer than that of post-ISM (Fig. 1f). The thermal energy above the 26 °C isotherm plays a critical role in the air-sea interaction and TC activities^14^. The region averaged 26 °C isothermal layer depth (D26) during pre- and post-ISM shows a difference of 5–10 m at times, while the mean difference remained 3.59 m (Fig. 1g). Further, it is also seen that the D26 has deepened 8.1 m and 8.5 m in pre- and post-ISM, respectively over the 41 years of analysis period (Table S2).

#### Ocean heat content and cyclone heat potential

**Fig. 2:**
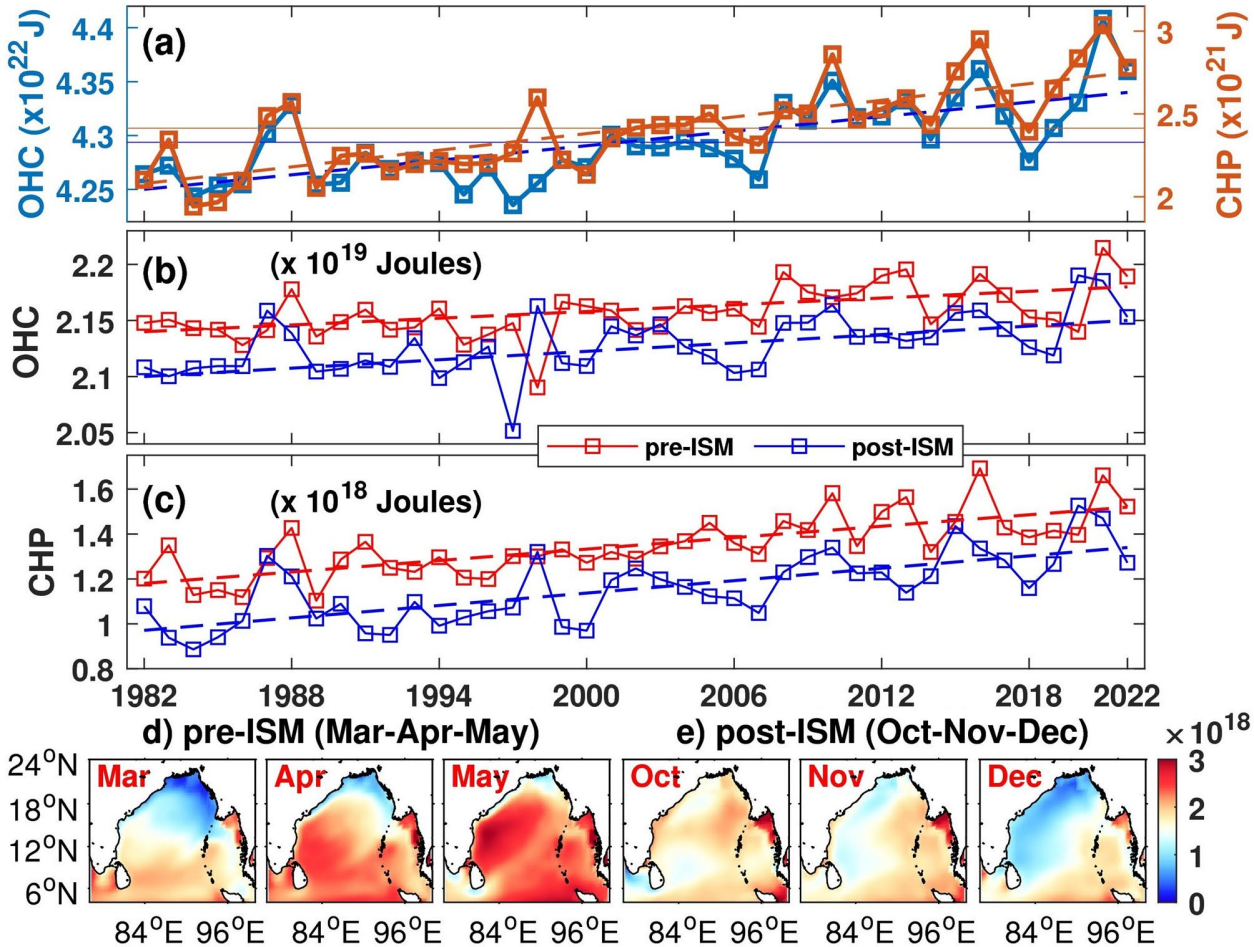
(**a**) Ocean Heat Content (OHC in Joules, blue line) of upper 100 m and Cyclone Heat Potential (CHP in Joules, brawn line) over the BoB. Same colour dotted and horizontal lines indicate the linear trend and mean of data, respectively. The OHC and CHP during pre- (red) and post-ISM (blue) with linear trend lines (same colour dotted lines) are shown in (**b**) and (**c**), respectively. The Spatial CHP pattern during pre- (March-April-May) and post-ISM (October-November-December) months are given in (**d**) and (**e**), respectively.

The OHC is the amount of heat energy stored in the volume of water. Higher OHC in the upper layers of the ocean can supply more energy to the atmosphere. Earlier study has reported that more than 4 × 10^4^ kJ/m^2^ of OHC can intensify the TCs in the BoB^26^. In view of the increasing surface and subsurface temperature, the OHC in the upper 100 m of the BoB is calculated and given in Fig. 2a, b. The annual mean OHC is seen 4.28×$\:{10}^{22}$ J and it has increased 9×$\:{10}^{20}$ J during 1982–2022 with a slope of 0.22×$\:{10}^{22}$ J/year (p-value 5.73×$\:{10}^{-14}$, Fig. 2a and Table S1). The seasonal mean OHC in pre-ISM (post-ISM) is seen 2.16×$\:{10}^{19}$ J (2.13×$\:{10}^{19}$ J, Fig. 2b and Table S2). The post-ISM OHC has shown higher dispersion (standard deviation 3×$\:{10}^{17}$ J) compared to the pre-ISM (standard deviation 2×$\:{10}^{17}$ J) and also accrued more heat (5×$\:{10}^{17}$ J) than the pre-ISM (4×$\:{10}^{17}$ J) during 1982-2022.

The CHP is a parameter to measure the available heat energy in the upper ocean above the 26 °C isotherm which is crucial for the energy supply to tropical cyclogenesis^14,18^. Now the increase in upper ocean temperature and OHC indicate that the CHP should also increase. The annual and seasonal average CHP are shown in Fig. 2a and c. As expected, the CHP shows an increasing pattern over these 41 years (Fig. 2a). The annual mean CHP is seen 2.41 ×$\:{10}^{21}$J and the CHP has increased 6.7×$\:{10}^{20}$ J with a slope of 1.69×$\:{10}^{19}$ J/year (p-value 2.1×$\:{10}^{-16}$, Table S1). The seasonal mean CHP remained high in pre-ISM (1.35×$\:{10}^{18}$ J, Fig. 2c) but similar to OHC, the CHP has increased comparatively more in post-ISM (3.7×$\:{10}^{17}$ J, Table S2). The special CHP pattern in pre-ISM shows a strong increase of CHP from March to May throughout the BoB (Fig. 2d). Whereas, a moderate CHP of 2×$\:{10}^{18}$ J (compared to > 3×$\:{10}^{18}$ J of pre-ISM) is seen in the southeastern part of the BoB during post-ISM (Fig. 2e).

#### Barrier layer thickness (BLT) and stability

**Fig. 3:**
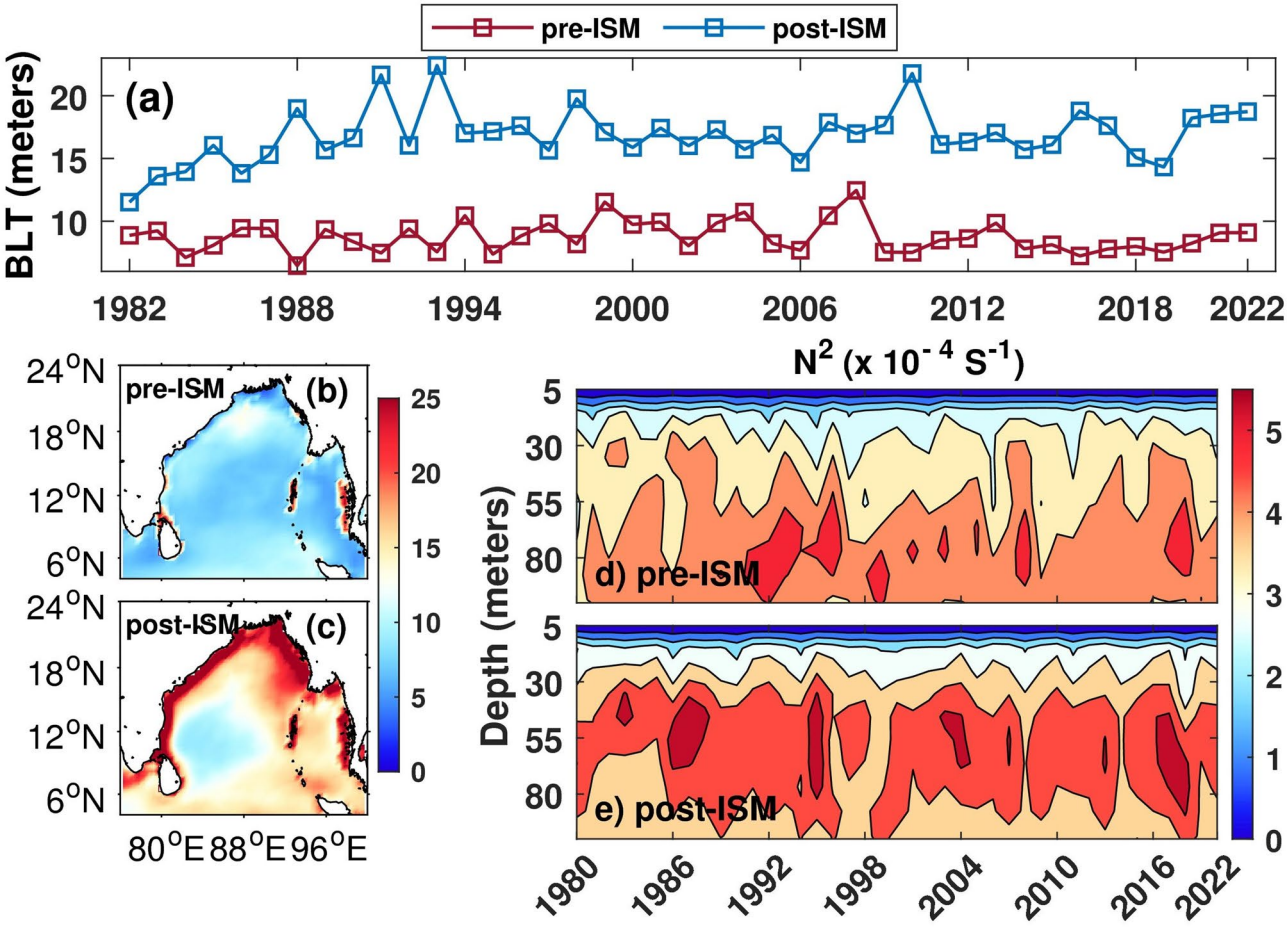
(**a**) Time series of Barrier Layer Thickness (BLT in meters) during pre- (red) and post-ISM (blue). The spatial pattern of BLT during pre- and post-ISM is given in (**b**) and (**c**), respectively. The vertical section of region averaged $\:{N}^{2}$ (square of Brunt-V$\:\ddot{\mathrm{a}}$is$\:\ddot{\mathrm{a}}$l$\:\ddot{\mathrm{a}}$ Frequency in $\:{s}^{-1}$) over the BoB with respect to depth during pre- and post-ISM is shown in (**d**) and (**e**), respectively.

The barrier layer is the difference between the isothermal layer and the mixed layer. In other words, it is the subsurface layer below the well mixed surface layer (called mixed layer) where temperature remained similar but the density increases due to increasing salinity. It occurs due to a shallow mixed layer compared to the isothermal layer. In the BoB, BLT formation happens mainly owing to the freshwater driven density stratification at the surface^27^. The barrier layer prevents the heat and momentum transfer between the mixed layer and the deep ocean, which in turn, stops the surface cooling and intensifies the tropical cyclones^18^. Moreover, the barrier layer helps the stratified mixed layer to retain the heat more proficiently which increases the mixed layer temperature. Here the BLT is calculated by the difference of the Mixed Layer Depth (MLD) based on density criteria from the MLD based on temperature criteria^28^. The BLT and associated vertical stability are given in Fig. 3.

The seasonal BLT during 1982–2022 is seen in between 5 and 12 m in pre-ISM, while it is 12–25 m in post-ISM (Fig. 3a). The seasonal mean BLT in pre-ISM is 8.75 m and the same is 16.84 m in post-ISM (Table S2). Further, the linear trend line of BLT shows that it is increased (decreased) 1.7 m (0.19 m) in post-ISM (pre-ISM) over the analysis period. The spatial BLT pattern shows that the BLT is ~ 5–10 m throughout the BoB during pre-ISM (Fig. 3b). In post-ISM, BLT is 15–20 m everywhere except the central-western BoB, where it is found relatively thinner (10–15 m, Fig. 3c).

The higher BLT is an indication of high vertical stratification which prevents vertical mixing (or surface cooling) and increases the vertical stability^29^. The salinity stratification in the BoB remains one of the important factors for seasonal differences in the oceanic response to air-sea thermal convections^3^. For measuring the vertical stability, the square of Brunt-V$\:\ddot{\mathrm{a}}$is$\:\ddot{\mathrm{a}}$l$\:\ddot{\mathrm{a}}$ Frequency ($\:{N}^{2}$) is calculated and shown in Fig. 3d, e. The region averaged $\:{N}^{2}$ section shows that the vertical stability is higher (~ 4.5×$\:{10}^{-4}$ $\:{s}^{-1}$) in the post-ISM compared to the pre-ISM (Fig. 3d, e). The vertical stability is stronger in the post-ISM particularly at 30–80 m depth level.

### Atmospheric forcing conditions

#### Outgoing heat fluxes

**Fig. 4:**
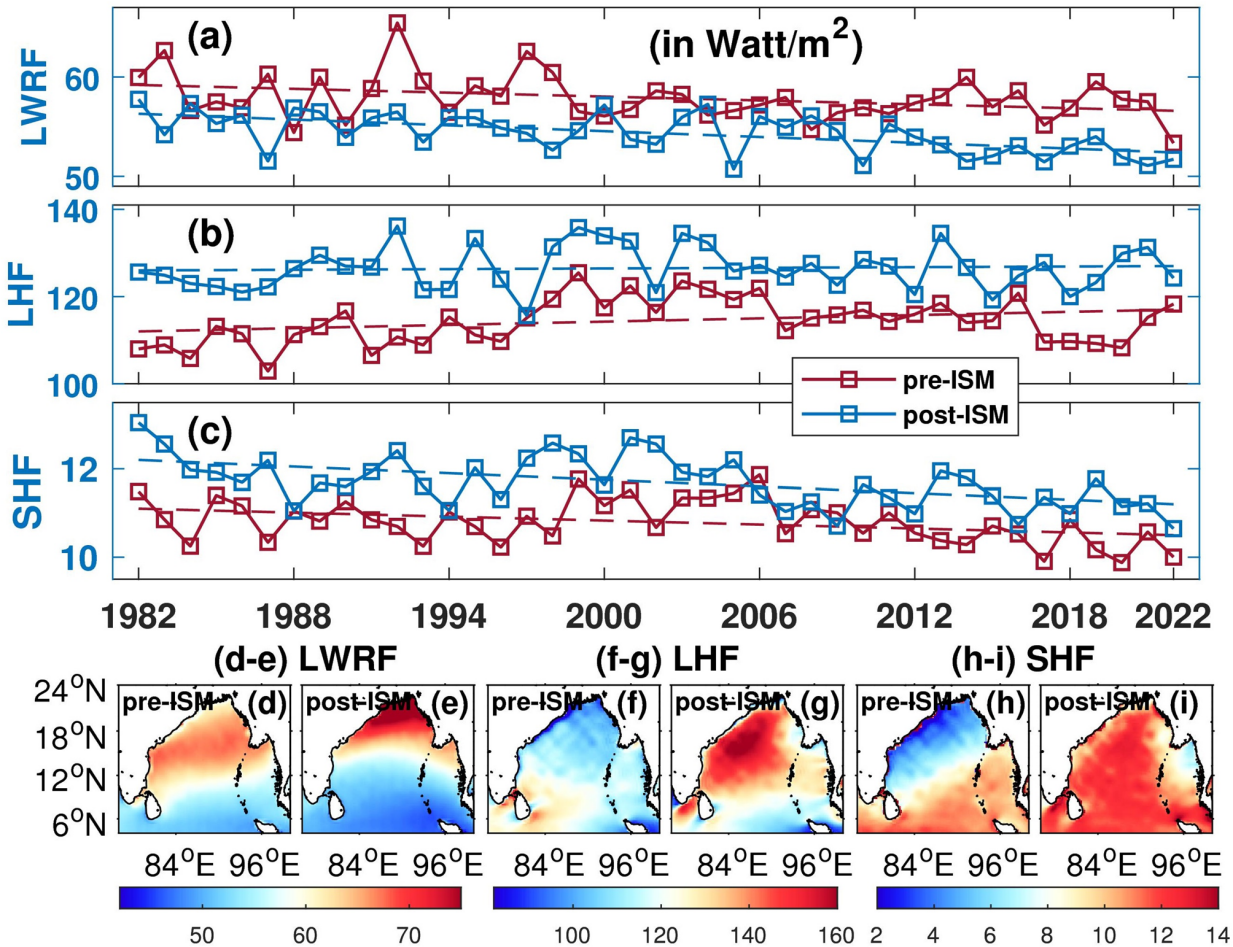
The time series of outgoing heat flux components (in W/m^2^) from the ocean, Long Wave Radiation Flux (LWRF), Latent Heat Flux (LHF) and Sensible Heat Flux (SHF) during pre- (red) and post-ISM (blue) are given in (**a**), (**b**) and (**c**), respectively. Dotted lines indicate the linear trend of corresponding data. Similarly, spatial pattern of LWRF, LHF and SHF during pre- and post-ISM are given in (**d**–**i**), respectively.

The outgoing heat fluxes (LWRF, SHF and LHF) from the ocean plays a crucial role for cyclogenesis and its intensifications. The cyclones generate and thrive on the heat transfer from ocean to the atmosphere through the heat fluxes. The spatiotemporal pattern of the outgoing heat flux components during pre- and post-ISM are given in Fig. 4 and the quantitative statistical values are given in Table S3 of the Supplementary Information.

The LWRF is the outgoing infrared radiative heat release from the ocean surface that influence the surface temperature and convection. Supressed LWRF is a favourable condition for increasing SST and cyclone activity^30^. The seasonal LWRF is seen 55–60 W/m^2^ (mean 57.89 W/m^2^) during pre-ISM, whereas it is 50–55 W/m^2^ (mean 54.31 W/m^2^) during post-ISM (Fig. 4a, Table S3). The linear trend of LWRF shows a decreasing tendency in both the seasons. During 1982–2022, the LWRF reduced 3.9 W/m^2^ in post-ISM (slope − 0.1 W/m^2^/year with p-value 6.18×$\:{10}^{-5}$), whereas the same reduction is 2.6 W/m^2^ in pre-ISM (slope − 0.063 W/m^2^/year with p-value 0.0382). The spatial pattern shows a north-south variation over the BoB (Fig. 4d, e). The LWRF is 60–70 W/m^2^ above 10°N in pre-ISM and 50–60 W/m^2^ below it. During post-ISM, the LWRF is mostly < 50 W/m^2^ except the northern top of the BoB, where it reaches > 70 W/m^2^.

LHF is the heat exchange from the surface ocean water to the atmosphere through the processes like evaporation and condensation. It primarily depends on wind speed, specific humidity, SST etc. The LHF plays most significant role in the energy feedback process to the cyclogenesis and its intensifications among the outgoing heat fluxes as the LHF supplies maximum energy from ocean to the atmosphere and influence air temperature, humidity, cloud formation and rain^31^. The seasonal LHF during 1982–2022 is seen higher in the post-ISM (120–140 W/m^2^ with mean 126.5 W/m^2^) due to high wind speed and low specific humidity compared to the pre-ISM (100–120 W/m^2^ with mean 114.3 W/m^2^, Fig. 4b and Table S3). The increasing rate of LHF in pre-ISM is higher (slope 0.14 W/m^2^/year with p-value 0.0439 and increased 5 W/m^2^) than that of post-ISM (slope 0.02 W/m^2^/year with p-value 0.768 and increased 1 W/m^2^). The spatial variation of seasonal LHF climatology in Fig. 4g shows that LHF is mostly very high over the BoB in the post-ISM (140–160 W/m^2^) with maximum value in the northwestern part (~ 160 W/m^2^). During pre-ISM, the LHF remains 100–120 W/m^2^ (Fig. 4f).

The SHF is the heat transfer from the ocean surface to the air through conduction and it mainly depend on surface air temperature and wind speed. Although the influence is less compared to other outgoing heat flux components, the direct heat transfer from ocean surface to atmosphere through SHF influences the surface air temperature and pressure^31^. Low air temperature and strong wind speed increases the seasonal SHF during post-ISM (11–13 W/m^2^ with mean 11.69 W/m^2^) than in pre-ISM (10–12 W/m^2^ with mean 10.8 W/m^2^, Fig. 4c). Further, the SHF shows a decreasing trend in both pre- and post-ISM reducing the heat flux 0.6–1 W/m^2^ (Table S3). Spatially, the SHF shows 12–15 W/m^2^ outgoing heat transfer throughout the BoB during post-ISM, while the same is 3–10 W/m^2^ in pre-ISM with minimum value in the northwestern BoB (3–6 W/m^2^, Fig. 4h, i).

#### Divergence of energy and moisture fluxes

**Fig. 5:**
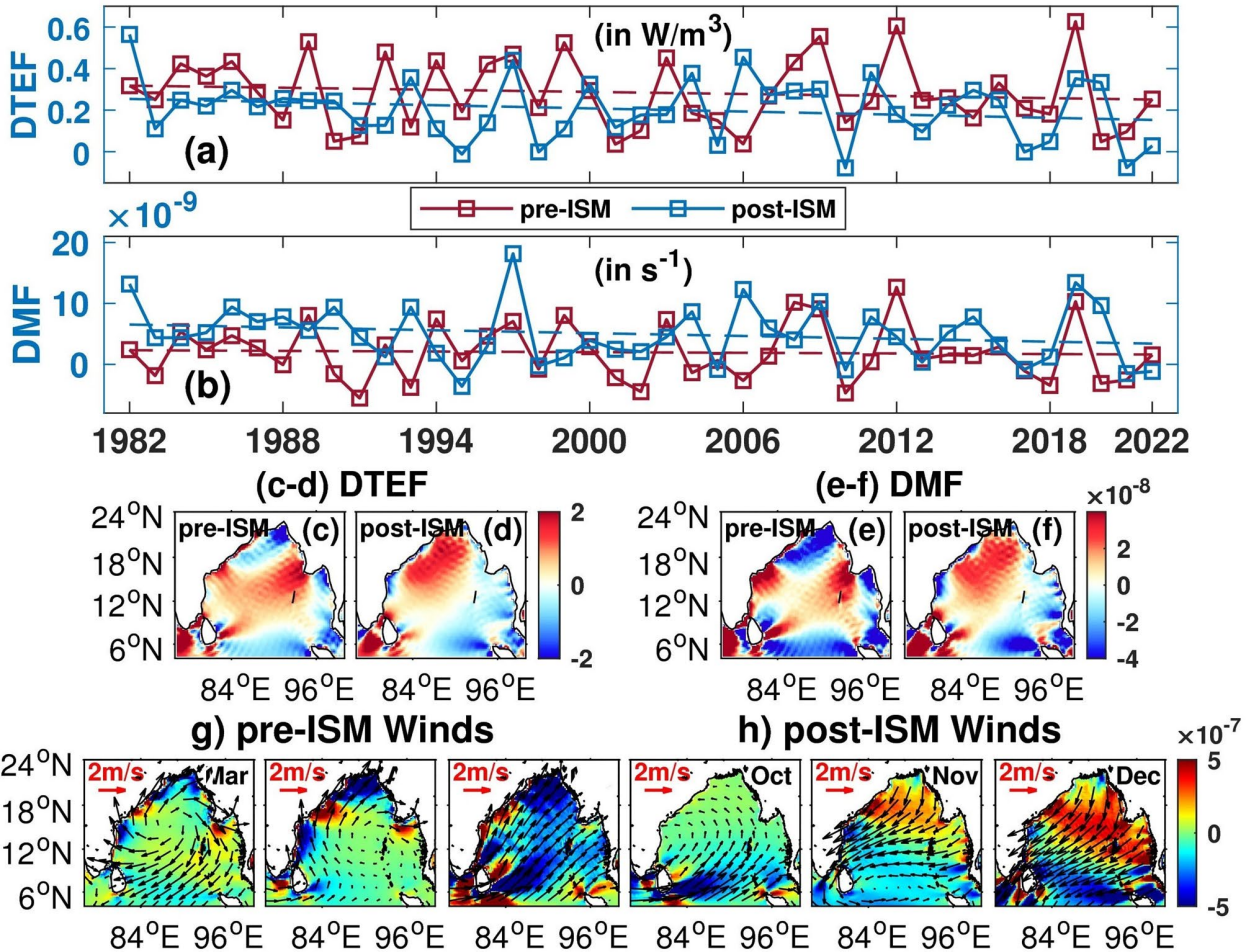
The Divergence of Thermal Energy Flux (DTEF in W/m^3^) and Divergence of Moisture Flux (DMF in s^− 1^) time series during pre- (red) and post-ISM (blue) are given in (**a**) and (**b**), respectively. The dotted lines denote the linear trend of data. The spatial DTEF and DMF patterns during pre- and post-ISM are given in (**c**,**d**) and (**e**,**f**), respectively. Monthly climatology of wind stress curl (in N/m^3^) along with wind vectors (in m/s at 10 m) during pre- (March-April-May) and post-ISM (October-November-December) months are given in (**g**) and (**h**), respectively.

The Divergence of Thermal Energy Flux (DTEF) essentially measures the outflow (positive) or inflow (negative) of available heat energy from a space in the atmosphere. Whereas, Divergence of Moisture Flux (DMF) is the measure of moisture being spreading out (positive) or concentrating in (negative) at the atmosphere over a place. Although the available atmospheric energy highly depends on temperature, the atmospheric moisture content has a dependency on temperature, evaporation, winds, precipitation, altitude etc. Both DTEF and DMF are crucial atmospheric parameters for tropical cyclogenesis and its intensifications. Lower tropospheric convergence of DTEF and DMF supplies energy and moisture to the depressions that favours cyclogenesis and its intensification^11,16^. The spatiotemporal patterns of seasonal DTEF and DMF averaged over 14 pressure levels between the pressure interval of 1000 − 600 hPa (see *Methods* section) are shown here in Fig. 5.

The seasonal mean DTEF in the period 1982 to 2022 shows that the average thermal energy divergence over the BoB is higher during pre-ISM (mean DTEF 0.29 W/m^3^) as compared to the post-ISM (mean DETF 0.204 W/m^3^, Fig. 5a and Table S3). In both the seasons, the DTEF has shown a slow decreasing trend over these 41 years. The energy divergence is relatively lower in post-ISM that makes this season more favourable for TC activities. Over the analysis period, the energy convergence is more increased during post-ISM with reducing DTEF (slope − 0.0025 W/m^3^/year and p-value 0.2). The DTEF is reduced 0.1 W/m^3^ in post-ISM compared to 0.067 W/m^3^ in pre-ISM during 1982–2022. The spatial pattern of seasonal DTEF climatology displays a strong thermal energy divergent over the northwestern BoB in post-ISM (> 1 W/m^3^, Fig. 5d) when the other parts mostly remain convergent. In pre-ISM, the central and central-western BoB shows a moderately high thermal energy divergent (1–2 W/m^3^, Fig. 5c).

The spatial pattern of seasonal DMF climatology is seen quite similar to the pattern of DTEF (Fig. 5e, f). In case of region average seasonal timeseries (Fig. 5b), the mean DMF is seen less in pre-ISM (1.96 × 10^− 9^ s^− 1^, Table S3) compared to post-ISM (4.96 × 10^− 9^ s^− 1^). This is due to the strong moisture convergence (< -2 × 10^− 8^ s^− 1^) in the northeastern top and southern BoB during pre-ISM (Fig. 5e). The seasonal mean DMF has shown a decreasing trend in both pre- and post-ISM (Fig. 5b). The mean DMF has reduced 3.13 × 10^− 9^ s^− 1^ in post-ISM (slope − 0.078 × 10^− 9^ s^− 1^/year with p-value 0.215) while this reduction is only 0.7 × 10^− 9^ s^− 1^ in pre-ISM (slope − 0.018 × 10^− 9^ s^− 1^/year) during 1982–2022. The spatial DMF pattern shows that the moisture concentration is very strong (convergent, <-2 × 10^− 8^ s^− 1^) in the southern and northwestern top of the BoB during pre-ISM, whereas the moisture is spreading out at the central and central-western BoB (Fig. 5e). In post-ISM, the DMF is highly positive in the northwestern BoB (> 2 × 10^− 8^ s^− 1^, Fig. 5f) similar to DETF.

#### Surface winds effect

The surface wind pattern plays a very important role in the genesis of a tropical cyclone. The inward wind flow over a low-pressure area favours the genesis of a tropical cyclone^23^. Further, the wind stress curl modulates the ocean subsurface thermal structure and 26 °C isotherm which then affects the tropical cyclone activities^9,14^. Here the monthly climatological wind directions with the wind stress curl during pre- and post-ISM months are given in Fig. 5g, h.

The surface wind pattern over the BoB shows that the March-April is the transition period when winds change the direction from northeasterlies to southwesterlies and remains low in magnitude (Mar-Apr, Fig. 5g). In May, the winds are relatively strong and the wind stress curl is mostly negative (< 2.5 × 10^− 7^ N/m^3^) over the BoB. In post-ISM, September and October are the transitions months for winds. During November-December, the winds are very strong southwest ward and the wind stress curl is positive (> 2 × 10^− 7^ N/m^3^) in northern and northeastern BoB (Nov-Dec, Fig. 5h).

### Long-term patterns (1982–2022) in tropical cyclone activities

#### Cyclone frequency

**Fig. 6:**
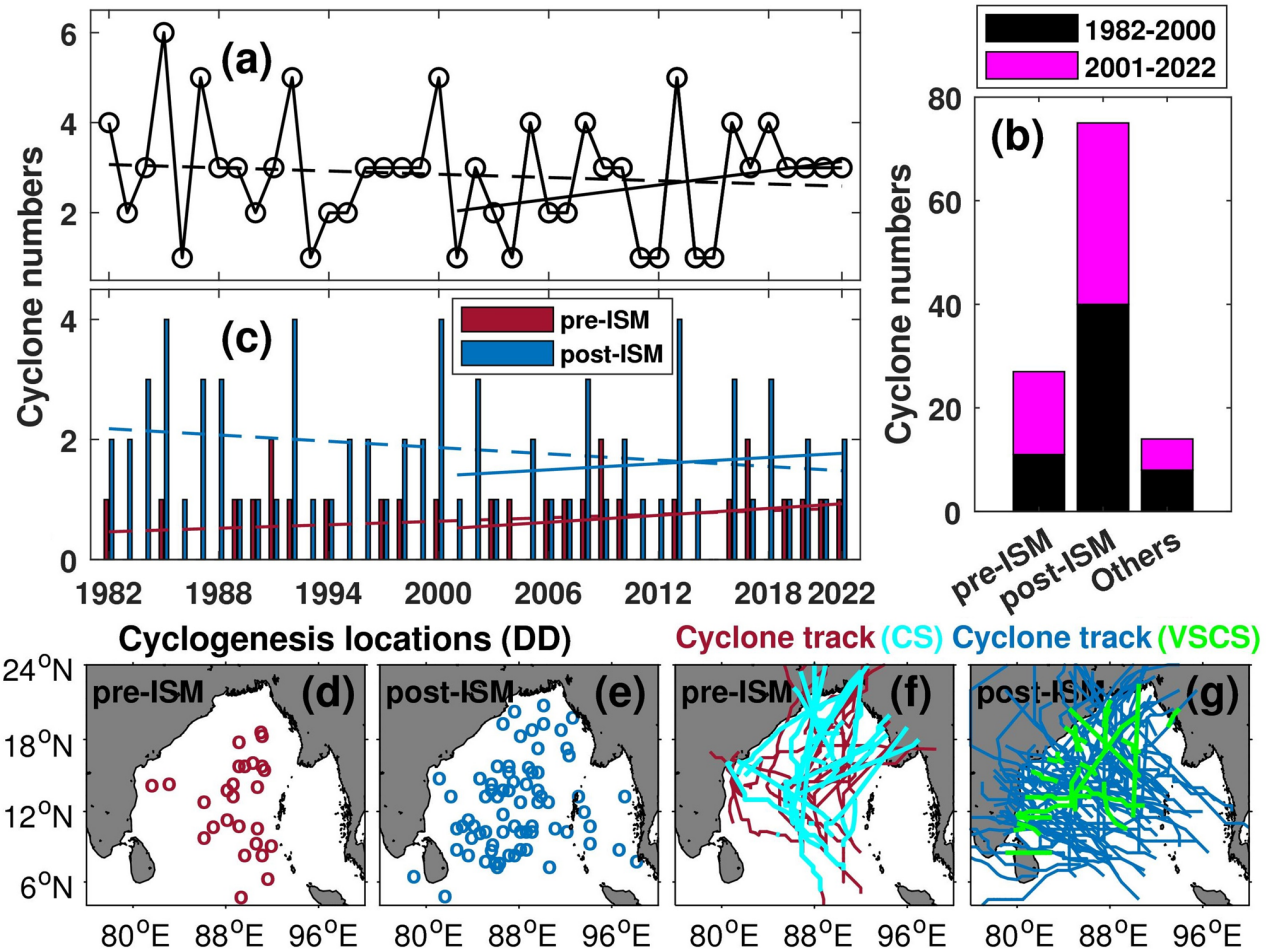
(**a**) Total number of yearly cyclones generated over the BOB during 1982–2022 with long term and recent linear trend (black dotted and solid lines, respectively). (**b**) Total number of seasonal cyclones occurred during 1982–2022 (1982–2000 in black and 2001–2022 in magenta). (**c**) Number of cyclones during pre- (red) and post-ISM (blue) with the long term and recent linear trends (dotted and solid lines, respectively). (**d**,**e**) Cyclogenesis points at DD stage during pre- (red) and post-ISM (blue). (**f**,**g**) Track of cyclones during pre- (red) and post-ISM (blue). The over imposed cyan and green tracks in (**f**) and (**g**) indicates the CS and VSCS activities during pre- and post-ISM, respectively.

During 1982–2022, total 116 low pressure systems were found over the BoB which reached to CS (Cyclonic Storm) or higher intensified category (see Methods section for all cyclone categories). Out of these 116 tropical cyclones, 27 occurred in pre-ISM (March-May, 23.3%) and 75 occurred in the post-ISM (October-December, 64.6%). Out of the remaining 14 cyclones (12.1%), 10 occurred during ISM (June-September) and the other 04 occurred during January-February (Fig. 6b). Earlier studies have reported that the strong southwestern monsoon winds and its robust vertical wind shear do not favour much cyclone to occur during ISM^32^. The annual cyclone frequency calculation considered the 116 cyclones, while the seasonal cyclone analysis used 27 pre-ISM and 75 post-ISM cyclones. The annual and post-ISM cyclone frequency over the BoB show a decreasing trend during 1982–2022 but this frequency is increasing for pre-ISM (Fig. 6a, c). The trend analysis shows that the overall annual cyclone frequency has reached from 3.07/year to 2.59/year in these 41 years with a mean of 2.83/year (Fig. 6a, Table S1). In post-ISM, the annual cyclone frequency has reduced from 2.18/year to 1.48/year (mean is 1.83/year), while the Pre-ISM shows the trend to rise from 0.46/year to 0.85/year (mean is 0.66/year, Fig. 6c, Table S4).

#### Cyclogenesis area and track

The locations of 26 pre-ISM and 76 post-ISM cyclones before intensifying into CS stage that is their DD (Deep Depression) stage locations are shown in Fig. 6d, e, respectively. Again, the tracks of these seasonal cyclones are given in Fig. 6f, g. The cyclone activities in CS stages (in cyan, Fig. 6f) during pre-ISM and VSCS (Very Severe Cyclonic Storm) stages (in green, Fig. 6g) during post-ISM are highlighted for the discussion in next *Cyclone intensity* section. The cyclogenesis locations show that the low-pressure systems, which are converting to CS or higher stages, are reaching their deep depression stage at the central and central-eastern part of the BoB during pre-ISM (Fig. 6d). Moreover, the tracks reveal that the cyclone activities are mainly spread out over the northern BoB and also most of the cyclone landfall occur towards the north of the BoB (Fig. 6f). In post-ISM, the cyclogenesis locations are mainly seen at the central and central-western part of the BoB (Fig. 6e). Further, the post-ISM cyclone activities are mainly distributed in the western part of the BoB with maximum landfall towards the west (Fig. 6g). The country wise cyclone landfall analysis in Table S5 shows that the landfall of pre-ISM cyclones during 1982–2022 occurs towards north of the BoB (Fig. 6f) in Bangladesh (BD, 44.44%), India (IN, 29.63%) and Myanmar (MN, 25.93%). Again, in India (Table S6), these pre-ISM landfalls are found only in three states, Andhra Pradesh (AP, 37.5%), Odissa (OD, 37.5%) and West Bengal (WB, 25%). As the cyclone genesis, movement and activities during post-ISM are mostly seen in the central and western BoB, maximum landfall also occurred in India (Fig. 6e, g). Out of 75 post-ISM cyclones during 1982–2022, 58 (77.33%) landed in IN, 11 landed in BD (14.67%) and the other 6 landfalls occurred in MN (5.33%) and SL (2.67%). In India, maximum post-ISM cyclone landfall occurred in AP (37.93%) followed by Tamil Nadu (TN, 34.48%), OD (15.52%), WB (10.34%) and Pondicherry (PY, 1.72%, Table S6).

#### Cyclone intensity

**Fig. 7:**
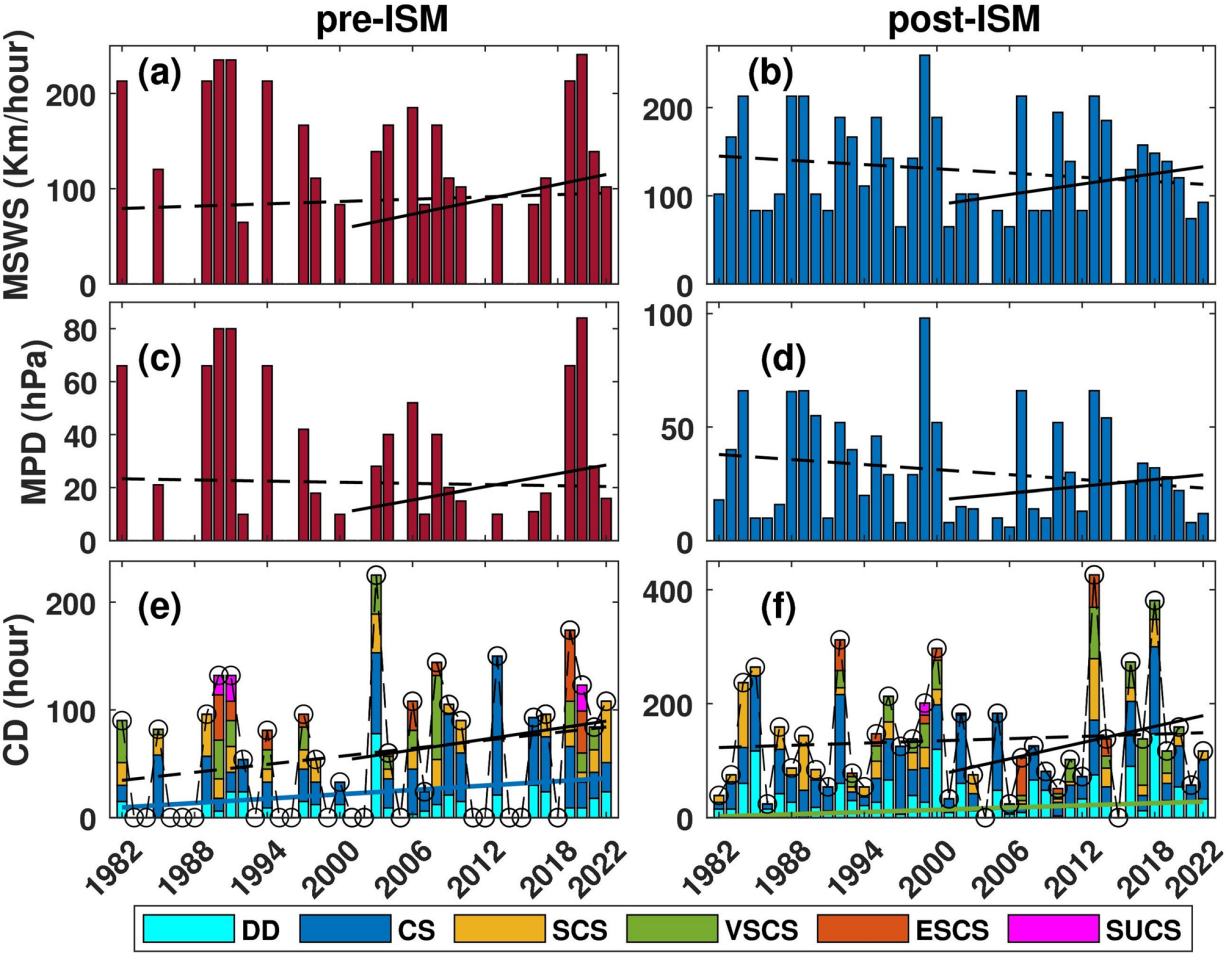
Maximum Sustained Wind Speed (MSWS in Km/hour, (**a**,**b**), Maximum Pressure Drop (MPD in hPa, (**c**,**d**) and Cyclone Durations (CD in hour, (**e**,**f**) of pre- (left column) and post-ISM (right column) cyclones during 1982–2022 with long-term and recent linear trends (black dotted and solid lines, respectively). The CD in (**e**) and (**f**) is given cyclone category wise. The blue and green solid lines in (**e**) and (**f**) are linear trends of the CD hours while the cyclone is in CS and VSCS stages, respectively.

The cyclone intensity during 1982–2022 is analysed based on three parameters, Maximum Sustained Wind Speed (MSWS), Maximum Pressure Drop (MPD) and Cyclone Duration (CD). The MSWS measures the unobstructed maximum wind speed observed (3 min average at 10 m height) while the cyclone is in its highest intensified category in-between CS to Super Cyclonic Storm (SUCS). Similarly, MPD measures the maximum pressure drop from normal atmospheric pressure, generally found when the cyclone is intensified to its peak. The CD is the number of total hours the cyclone activity lasted from its DD formation stage to DD dissipation stage. The seasonal mean MSWS, MPD and CD during 1982–2022 are given in Fig. 7 and associated statistics are included in Table S4.

**Fig. 8:**
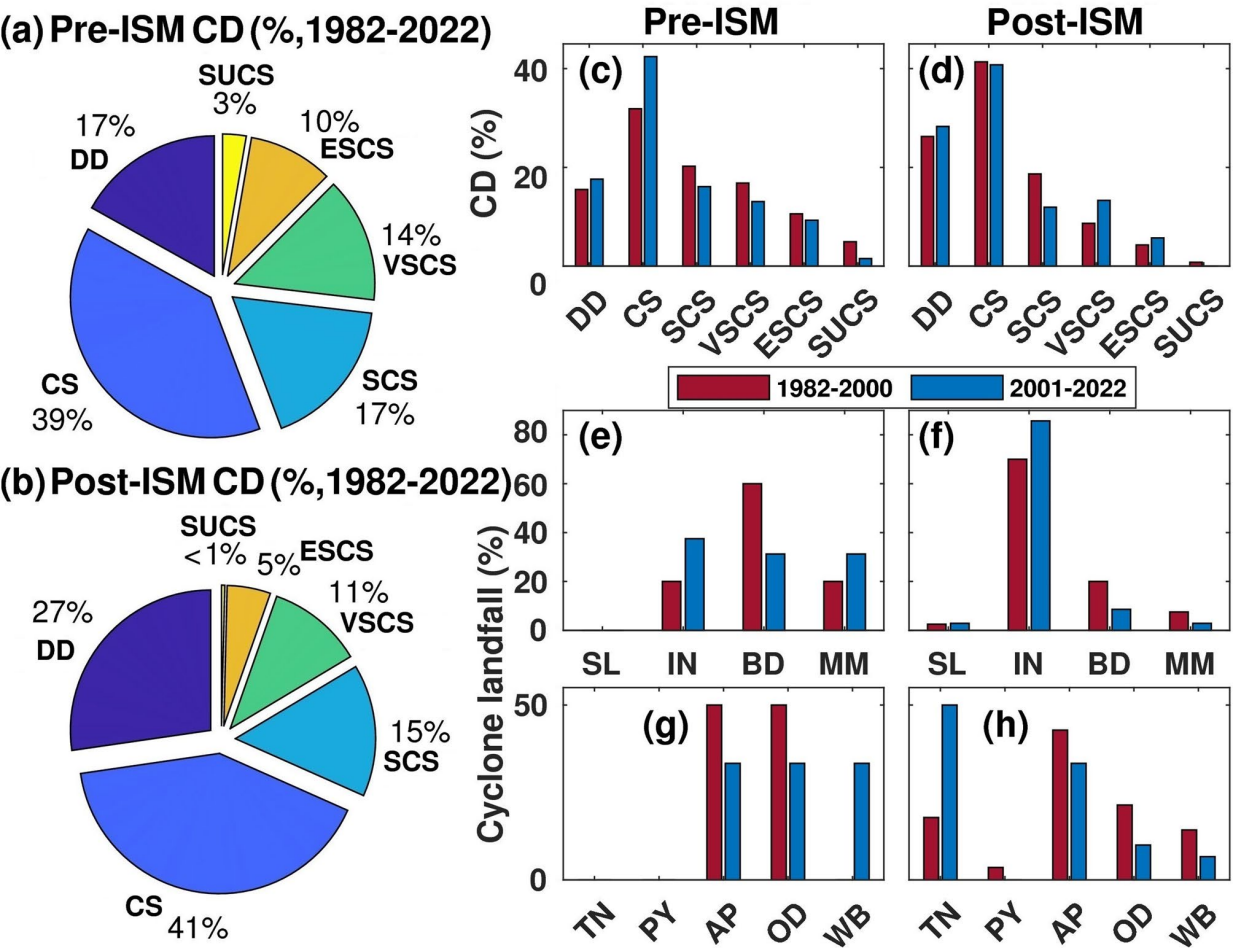
(**a**,**b**) Cyclone category wise total CD hours (in %) during 1982–2022 of pre- (in **a**) and post-ISM (in **b**) cyclones. (**c**,**d**) Similar to (**a**,**b**), cyclone category wise total CD hours (in %) for pre- and post-ISM cyclones during 1982–2000 (in **c**) and 2001–2022 (in **d**). (**e**,**f**) Country wise cyclone landfall percentages (%) of the BoB cyclones. (**g**,**h**) Among the cyclones which made landfall in India, the Indian coastal state wise landfall percentages (%). SL = Sri Lanka, IN = India, BD = Bangladesh, MM = Myanmar, TN = Tamil Nadu, PY = Pondicherry, AP = Andhra Pradesh; OD = Odissa, WB = West Bengal.

The seasonal mean MSWS in pre- and post-ISM is seen respectively 87.36 Km/h and 129 Km/h (Table S4). Over the analysis period, the mean MSWS is seen increasing during pre-ISM while a decreasing trend is noticed in post-ISM (Fig. 7a, b). The mean MSWS has increased 16.2 Km/h during pre-ISM of 1982–2022 and reduced 32 Km/h in the post-ISM. The seasonal mean MPD is found 21.88 hPa during pre-ISM and 30.5 hPa for post-ISM (Fig. 7c, d). In consistent with the decreasing MSWS, MPD is also seen decreasing during post-ISM (slope − 0.37 hPa/year with p-value 0.234) and reduces the mean pressure fall up to 14.8 hPa (Table S4). In pre-ISM, the MPD is seen to be decreasing very slowly (slope − 0.073 hPa/year and reduces 2.9 hPa in 41 years). Even though MPD showed a weak decreasing trend during pre-ISM of 1982–2022, the MPD and MSWS exhibit opposite trends as seen in the region average timeseries data (Fig. 7a, c). This might be due to various reasons like TC size, TC genesis latitude, translation speed or intensification phase^33^. The CD pattern has shown a sharp rise during pre-ISM (slope 1.24 h/year with p-value 0.124) and increased the average CD value 49.5 h over 1982–2022 while the mean remains 59.37 h. On the other hand, after having a higher mean also (135.9 h), the CD has increased in a relatively slower rate (slope 0.67 h/year, increased 26 h in 41 years) during the post-ISM (Fig. 7e, f). Over the 41 years of analysis period, the maximum increase in CD is seen when the TC is in CS category during pre-ISM and in VSCS category during post-ISM (Fig. 7e, f). Tracks of such TCs (CS during pre-ISM and VSCS during post-ISM) are highlighted by cyan and green in Fig. 6e, f. It is seen that, the average pre-ISM CD has increased 27 h during 1982–2022 when the TC is in CS category (Fig. 7e, blue trend line). Similarly, the post-ISM CD in these years has seen a rise of 26 h when the TC is in VSCS stage (Fig. 7f, green trend line). As the seasonal CD pattern is showing a substantial increase during different TC category over the BoB, this parameter is further analysed in Fig. 8a–d according to the cyclone categories. While Fig. 8c, d reveal the recent and previous changes in CD and to be discussed in the next section, Fig. 8a, b show that on an average how much time (CD) a TC sustain in each stage of cyclone intensification during pre- and post-ISM of 1982–2022. The Fig. 8a reveals that the cyclones do not stay in DD stage for much time (17%) before intensifying to the CS or higher intensified stages during pre-ISM. Again, on an average pre-ISM cyclone sustains maximum time in CS stage (39%) and the intensification has reached up to SUCS stage (3%). On the contrary, the post-ISM cyclones stay much more time in the DD stage (27%) before further intensification and the chances to reach to SUCS stage is very less (< 1%, Fig. 8b). Therefore, although the pre-ISM cyclones are short lived compared to post-ISM cyclones, the pre-ISM cyclones intensify in much faster rate than that of post-ISM cyclones.

## Discussions

### Upper-ocean and atmospheric influences on tropical cyclone activities

In the pre-ISM, very high surface (> 29 °C, Fig. 1e) and subsurface (> 27.5 °C, Fig. 1f) ocean temperature in the central and central-eastern BoB favours the cyclogenesis there (Fig. 6d). This high temperature and its strong rising rate at surface (0.017 °C/year, Table S2) during 1982–2022 leads to a high OHC (> 2.16 × 10^19^ J, Table S2). Again, the high OHC and deeper D26 make the CHP substantially high (> 2 × 10^18^ J, Fig. 2d). Thus, the warmer upper ocean supplies more energy and moisture to the atmosphere which increases the sustainability as well as the rise in intensity in the TC activities during pre-ISM. Energy and moisture convergence in the southeastern BoB helps the TC genesis there (Fig. 5c, e). The wind direction helps the northward propagation and activities of TCs (Figs. 5g and 6f).

During post-ISM, very thick barrier layer (> 15 m, Fig. 3a, c) and subsequent strong vertical stratification (N^2^ > 4.5 × 10^− 4^ s^− 1^, Fig. 3e) largely reduce the surface cooling^34^ and increases the convective heat release to the atmosphere (Fig. 4b, c,g, i). The energy and moisture convergence at the central-western BoB (Fig. 5d, f) mostly influence the TC activities there (Fig. 6e). The wind directions and the strong positive wild stress curl help the TC propagation and activities in the central and western BoB (Figs. 5h and 6g).

### Recent patterns (2001–2022) in air-sea conditions and cyclone activities

A substantial change in the patterns of TC activities and also in overall air-sea conditions after the year 2000 is observed from the analysis. Similar mention can also be found in earlier studies where it is reported that the TC intensifications have increased rapidly after 2000 over the NIO as well as the BoB^7,8^. This section highlights the differences in pattern of the upper ocean parameters, atmospheric variables and TC activities between the periods 1982–2000 (19 years, also referred as earlier period) and 2001–2022 (22 years, also referred as recent period) based on the quantitative statistical analysis given in Tables S2-S6. Some of the recent trends and analysis for TC activities are also included in Figs. 6, 7 and 8.

The SST and SubST of the BoB have shown a steady increase during pre- and post-ISM of 2001–2022 compared to 1982–2000 (Table S2). During pre- and post-ISM of 1982–2000, the mean SST over the BoB has shown either no increment (post-ISM) or marginal increment (0.1 °C in pre-ISM), But during 2001–2022, the SST has increased 0.3 °C (post-ISM) to 0.5 °C (pre-ISM). For SubST, the post-ISM shows relatively more increase than the pre-ISM. The subsurface temperature has either decreased (0.1 °C in pre-ISM) or remained same (in post-ISM) during 1982–2000. Conversely, 0.3 °C (pre-ISM) to 0.4 °C (post-ISM) SubST increase is noticed during 2001–2022. The mean D26 is deepened substantially (6.1–7.1 m) in the recent years from almost constant mean D26 of earlier period (increased 0–0.5 m). It is discussed previously that the BLT has changed minorly during the analysis period of 1982–2022 (~ 2 m, Table S2). A similar pattern is also seen in recent period (2001–2022). Whereas, the earlier period shows an increase of BLT during both pre- and post-ISM (1.34 m and 4.4 m, respectively). The OHC in both pre- (2 × 10^17^ J) and post-ISM (3 × 10^17^ J) has seen a good amount of increase during 2001–2022 compared to 1982–2000 when the OHC is seen to reduce (pre-ISM) or remain unchanged (post-ISM). Although the CHP has shown an increasing pattern in both earlier and recent periods, the rate of increase is more in recent period and also in the pre-ISM.

The outgoing LWRF has a decreasing trend during both pre- and post-ISM over 1982–2000 as well as 2001–2022 (Tables S3). In pre-ISM, the decreasing rate of LWRF seems to have a similar slope during earlier (-0.018 W/m^2^/year) and recent (-0.014 W/m^2^/year) periods. But post-ISM retained more heat in the recent period (3.3 W/m^2^) compared to the earlier period (1.2 W/m^2^). The LHF shows a contrasting pattern during 1982–2000 and 2001–2022. In the earlier period, the LHF has an increasing trend resulting 11 W/m^2^ and 7 W/m^2^ increase during pre- and post-ISM, respectively. But a decrease in LHF of 8 W/m^2^ and 3 W/m^2^ are seen during pre- and post-ISM of the recent period. The SHF has decreasing trend over the analysis period (1982–2022) that reduces the SHF 0-0.3 W/m^2^ in earlier period and 1.1–1.3 W/m^2^ in recent period. The DTEF and DMF have a decreasing pattern in post-ISM during both earlier as well as recent period. The post-ISM DTEF has reduced with a slope of -0.0081 W/m^3^/year (p-value 0.184) during 1982–2000 when the decreased energy divergence is 0.15 W/m^3^. In the recent period (2001–2022), the post-ISM DTEF has decreased 0.112 W/m^3^ with a slope of -0.0053 W/m^3^/year (p-value 0.314). In pre-ISM, the DTEF has increased over the entire analysis period that increases the energy divergence 0.067 W/m^3^ (0.052 W/m^3^) during 1982–2000 (2001–2022). The seasonal DMF shows similar trend (sign) like DTEF over the earlier and recent times. The DMF has reduced 4.95 × 10^− 9^ s^− 1^ and 1.51 × 10^− 9^ s^− 1^ in the post-ISM of 1982–2000 and 2001–2022, respectively. In pre-ISM, the DMF has increased by 1.99 × 10^− 9^ s^− 1^ and 0.25 × 10^− 9^ s^− 1^ over the earlier and recent times, respectively.

The TC activity (Table S4, Figs. 6, 7 and 8) and landfall (Tables S5-6, Fig. 8) statistics have seen to be modified a lot in the recent period. The overall TC frequency shows a steady increase during 2001–2022 (Fig. 6a, solid black trend line). During 1982–2000, the mean TC frequency over the BoB was 0.58 and 2.1 during pre- and post-ISM, respectively. The mean TC frequency value has increased (decreased) up to 0.25 (0.29) during pre-ISM (post-ISM, Table S4) in the earlier period. On the other side, the mean TC frequency has increased in both the seasons during 2001–2022 (Fig. 6c). The mean TC frequency of 0.73 and 1.59 have seen an increment of 0.403 and 0.36 during pre- and post-ISM, respectively. In the TC intensifications during 1982–2000, the MSWS has reduces marginally during pre-ISM (2.1 Km/h) while a sharp increase is noticed during post-ISM (40 Km/h). During 2001–2022, the MSWS has increased rapidly (Fig. 7a, b) during both pre- (slope 2.6, increased 54.8 Km/h) and post-ISM (slope 1.97, increased 41.3 Km/h). Although the mean MPD has reduced (8.1 hPa) in pre-ISM and risen (16.5 hPa) in post-ISM during earlier period, the recent period has shown a steady increase in both the seasons (17.2 and 10.5 hPa during pre- and post-ISM, respectively, Fig. 7c, d). Similar to MPD, the mean CD also shows a low reduction (2.5 h) in the pre-ISM of 1982–2000, while the post-ISM has increased the mean CD up to 85 h. In recent period, the mean CD has increased in both pre- (35.2 h) and post-ISM (99.6 h, Table S4). While the rise in mean CD is steady in pre-ISM, the post-ISM increase owes more to the years 2012 and 2018 (Fig. 7e, f). The CD percentage analysis in Fig. 8c, d shows that the TC activity has increased the mean CD during pre-ISM of the recent period when the cyclone remains in CS category. The CD of TC categories higher than CS have reduced marginally in this time period. During post-ISM, the mean CD has reduced for TC category SCS but increased for VSCS in the recent period.

The landfall percentage of pre-ISM cyclones have increased in IN by 19.32%, MN by 13.07% and decreased in BD by 32.39% from earlier period to the recent period (Fig. 8e, Table S5). Moreover, in post-ISM, the TC landfalls have reduced in BD (by 11.43%), MN (by 4.64%) and increased in IN (by 15.71%, Fig. 8f). This TC landfall increase in IN has reflected on the Indian state wise statistics where the post-ISM landfall increased in TN (by 32.14%) but decreased in other states (AP, OD, WB) from earlier period to recent period (Fig. 8h, Table S6). In pre-ISM, the TC landfall increased in WB and reduced in AP and OD in recent times (Fig. 8g).

Although the topic needs more long-term observational data analysis between air-sea feedback and TC activities, this study observed a major shift in the upper ocean temperature and associated thermodynamic parameters (like D26, OHC, CHP) over the BoB in the recent period (after the year 2000). Figure 1a shows that the rising surface and subsurface temperature is above temporal average in recent times, when the average D26 also becomes deeper (Fig. 1d). The OHC and CHP also have the similar recent patterns as that of the temperature (Fig. 2a). The strong radiative heat fluxes backed by the high valued upper ocean thermodynamic parameters facilitate the strong moisture and energy convergence at the southeastern and southern BoB to influence the genesis and activities of the TCs (Figs. 4d–i and 5c–f). Moreover, the TC frequency and intensity have increased over the BoB in the recent period during both pre- and post-ISM (Figs. 6c and 7) in which Indian coastline is found to be the most vulnerable in general (Fig. 8e–h).

## Conclusions

The study presents a quantitative analysis and discussions on the seasonality of upper ocean thermodynamic conditions, related atmospheric variables and the TC activities over the BoB during pre- and post-ISM. For the analysis the study used SODA3.15.2 ocean data, ECMWF ERA5 atmospheric data and IMD cyclone data during 1982–2022. The study also highlights the changes in the pattern of air-sea conditions and TC activities in recent years (2001–2022).

In the upper ocean thermodynamics, the surface temperature has shown a higher rising trend during pre-ISM while the subsurface heat accumulation is seen higher in the post-ISM. Moreover, the recent years have contributed the majority of the enhanced temperature, associated OHC and CHP. The D26 has seen a substantial deepening in the recent years. The thick BLT remained instrumental for strong stratification and reduces surface cooling in the post-ISM. Among the outgoing heat fluxes, suppressed LWRF condition remained influential in the post-ISM specially in the recent years. The variation in LHF is seen dominant in pre-ISM but the pattern remained contrasting with an increase (decrease) during 1982–2000 (2001–2022). The SHF remains relatively high in the post-ISM due to the stronger winds. The atmospheric thermal energy flux remains more favourable for TC activities during the post-ISM (DTEF reduced), whereas the pre-ISM has seen an increase in energy divergent. The atmospheric moisture follows very much similar pattern of the energy flux.

Although the overall cyclone frequency has a decreasing pattern, the seasonal analysis reflects another picture. The pre-ISM always showed an increasing TC frequency, while the post-ISM TC frequency is seen increasing in recent years. In addition to this, all TC intensification parameters have shown an increasing trend in recent times and the percentage of increase is mostly steady and high in pre-ISM except the CD that too due to the dominance of 2013 and 2018. The TC landfalls during 1982–2022 have shown a northward inclination towards BD in pre-ISM, although IN and MN also have good share. The changing landfall pattern, in recent years, shows a sizable rise of the TC movement towards IN and MN during pre-ISM. In post-ISM, the TC movement mostly remained west and northwest ward towards IN followed by BD and MN. In recent years, the westward movement of post-ISM TCs has increased substantially mostly affecting the Indian states of TN. The winds pattern plays a critical role in TC movement.

Overall, the study suggests that the air-sea conditions with seasonal variations remained very much favourable for the increased and intensified TC activities over the BoB in recent years. While the BLT, DTEF, DMF, LWRF, SHF, wind stress curl remained pivotal for intensified TC activities during post-ISM in recent times, the pre-ISM TC intensifications are largely fuelled by high OHC, CHP and LHF above the D26. Furthermore, patterns in TC activities have shown a major shift during 2001–2022 in terms of intensity and landfall. Although the topic needs more observational studies for better understanding of the air-sea feedback and TC activities in higher scale, this quantitative analysis can play an important part in future studies and will also be helpful in estimating the air-sea as well as TC parameters.

## Methods

The upper ocean is considered 0–100 m for evaluating oceanic parameters. The subsurface analysis is done by taking the depth averaged value in 0–100 m depth. The months taken in pre- and post-ISM seasons are March-April-May and October-November-December, respectively. The low pressure systems are categorized in to seven different types based on the intensity (wind speed) as provided by Indian Meteorological Department (IMD) namely, Depression (D, 31–49 km/h), Deep Depression (DD, 50–61 km/h), Cyclonic Storm (CS, 62–88 km/h), Severe Cyclonic Storm (SCS, 89–117 km/h), Very Severe Cyclonic Storm (VSCS, 118–166 km/h), Extremely Severe Cyclonic Storm (ESCS, 167–221 km/h) and Super Cyclonic Storm (SUCS, > 222 km/h). This study mostly discussed the categories from DD to SUCS in the analysis and results.

The tables (Tables S1–S6) of quantitative analysis listing the details statistical variations of the ocean parameters (Tables S1-2), atmospheric parameters (Table S3), TC parameters (Table S4) and TC landfall (Tables S5-6, country and Indian state wise) are included in the Supplementary Information.

### Ocean heat content (OHC)

The OHC in the upper 100 m of the BoB is calculated using the following Eq. 

1 $$\:OHC={\rho\:}_{0}{C}_{p}A{\int\:}_{100\:m\:depth}^{surface}Tdz$$

Here, $\:{\rho\:}_{0}$ is the ocean water density (1025 kg/m^3^), $\:{C}_{p}$ is the specific heat of ocean water (4 $\:kJ/kg.^\circ\:C$), *A* is the horizontal grid area of temperature data, $\:T$ is the ocean temperature and $\:dz$ is the vertical grid length.

### Cyclone heat potential (CHP)

The CHP over the BoB is calculated using the formula given below.

2 $$\:CHP={{\rho\:}_{0}C}_{p}A{\int\:}_{depth\:(T=26^\circ\:C)}^{surface}(T-26)dz$$

While all the terms of Eq. (2) are defined in Eq. (1), the only difference here is that the depth integration of temperature ($\:T-26^\circ\:C$) is required from surface to D26.

### Barrier layer thickness (BLT)

The BLT is calculated by using the formula,

$$\:BLT=Isothermal\:layer\:depth\:\left(ILD\right)-Mixed\:layer\:depth\:\left(MLD\right)$$

3 $$=MLD\:from\:temperature\:criteria-MLD\:from\:density\:criteria$$

The MLD from temperature/density criteria finds the water depth from surface where the temperature/density differ from the surface value by a specific threshold value (generally 0.5°-0.8 °C/0.03–0.05 kg m^− 3^).

### Brunt-V$\:\ddot{{a}}$is$\:\ddot{{a}}$l$\:\ddot{{a}}$ frequency (BVF)

The square of BVF ($\:{N}^{2}$) can be calculated by using the equation given below.

4 $$\:{N}^{2}=\frac{g}{{\rho\:}_{0}}\frac{d\rho\:}{dz}$$

Where, $\:g$ is acceleration due to gravity (9.81 $\:m/{s}^{2}$), $\:\rho\:$ is potential density of ocean water and $\:z$ is ocean water depth.

### Divergence of thermal energy flux (DTEF)

The horizontal DTEF averaged over 14 lower atmospheric pressure levels (1000, 975, 950, 925, 900, 875, 850, 825, 800, 775, 750, 700, 650, 600 hPa) is calculated by using the formula given below.

5 $$\:DTEF={\rho\:}_{air}{C}_{p\left(air\right)}\nabla\:.\left(t\stackrel{-}{V}\right)$$

Here, the $\:{\rho\:}_{air}$ is air density (1.225 kg/m^3^), $\:{C}_{p\left(air\right)}$ is specific heat of air (1.005 $\:kJ/Kg.^\circ\:C$), $\:t$ is air temperature at the pressure levels and $\:\stackrel{-}{V}=\stackrel{-}{V}(u,v)$ are the horizontal wind velocity components ($\:u$ is zonal component and $\:v$ is meridional component) at pressure levels.

### Divergence of moisture flux (DMF)

The average DMF (over 14 pressure levels between 1000 − 600 hPa as given similar to DTEF) can be calculated by using the formula,

6 $$\:DMF=\nabla\:.\left(q\stackrel{-}{V}\right)$$

where, $\:q$ is the specific humidity at the pressure levels and $\:\stackrel{-}{V}$ is horizontal wind velocity as given in Eq. (5).

## Supplementary Information

Below is the link to the electronic supplementary material.

Supplementary Material 1

## Data Availability

*Temperature and MLD*: The BoB temperature and MLD datasets are used from Simple Ocean Data Assimilation ocean/ sea ice analysis (SODA3.15.2). The SODA3.15.2 used the atmospheric forcing from the fifth generation ECMWF (European Centre for Medium-Range Weather Forecasts) reanalysis for global climate and weather (ERA5). The data resolution is levels in a Mercator horizontal uniform grid. The data is available at https://apdrc.soest.hawaii.edu/. *Heat fluxes, specific humidity, air temperature and winds*: The study used the data of outgoing heat flux components (LWRF, LHF and SHF), specific humidity, atmospheric temperature and winds from ECMWF ERA5. It is a reanalysis product which combines model and observational datasets to make a globally complete and consistent data which replaced the earlier ERA interim datasets. The horizontal data resolution is in a regular uniform grid. The datasets are available at https://cds.climate.copernicus.eu/datasets. *Cyclones*: The complete tropical cyclone activity data over the BoB is provided by Indian Meteorological Department (IMD). The cyclone best track data is available at https://rsmcnewdelhi.imd.gov.in/index.php.

## References

[1] Prasad, T. G. A comparison of mixed-layer dynamics between the Arabian Sea and Bay of Bengal: One‐dimensional model results. *JGR: Oceans*. **109**(C3) (2004).

[2] Phillips, H. E. et al. Progress in understanding of Indian Ocean circulation, variability, air–sea exchange, and impacts on biogeochemistry. *Ocean. Sci.* **17**(6), 1677–1751 (2021).

[3] Singh, V. K. & Roxy, M. K. A review of ocean-atmosphere interactions during tropical cyclones in the north Indian Ocean. *Earth-Sci. Rev.* **226**, 103967 (2022).

[4] Samarakoon, J. Issues of livelihood, sustainable development, and governance: Bay of Bengal. *Amibo* **33**(1), 34–44 (2004). 15083648

[5] Anandh, T. S., Das, B. K., Kumar, B., Kuttippurath, J. & Chakraborty, A. Analyses of the oceanic heat content during 1980–2014 and satellite-era cyclones over Bay of Bengal. *Int. J. Climatol*. **38**(15), 5619–5632 (2018).

[6] Balaji, M., Chakraborty, A. & Mandal, M. Changes in tropical cyclone activity in north Indian Ocean during satellite era (1981–2014). *Int. J. Climatol*. **38**(6), 2819–2837 (2018).

[7] Nadimpalli, R. et al. Understanding the characteristics of rapid intensity changes of Tropical Cyclones over North Indian Ocean. *Discov appl. Sci.* **3**(1), 68 (2021).

[8] Deshpande, M. et al. Changing status of tropical cyclones over the north Indian Ocean. *Clim. Dyn.* **57**(11), 3545–3567 (2021).

[9] Neetu, S. et al. Premonsoon/postmonsoon Bay of Bengal tropical cyclones intensity: Role of air-sea coupling and large‐scale background state. *Geophys. Res. Lett.* **46**(4), 2149–2157 (2019).

[10] Priya, P., Pattnaik, S. & Trivedi, D. Characteristics of the tropical cyclones over the North Indian Ocean Basins from the long-term datasets. *Meteorol. Atmos. Phys.* **134**(4), 65 (2022).

[11] Jena, B. & Pattnaik, S. Interdecadal variability of the pre-monsoon cyclone characteristics over the Bay of Bengal. *Environ. res. Clim.* **3**(2), 025003 (2024).

[12] Mohapatra, M., Sharma, M., Devi, S. S., Kumar, S. V. J. & Sabade, B. S. Frequency of genesis and landfall of different categories of tropical cyclones over the North Indian Ocean. *Mausam* **72**(1), 1–26 (2021).

[13] Mondal, M. et al. Spatio-temporal behaviours of tropical cyclones over the Bay of Bengal Basin in last five decades. *Trop. Cyclone Res. Rev.* **11**(1), 1–15 (2022).

[14] Gao, C. et al. Crucial role of subsurface ocean variability in tropical cyclone genesis. *Nat. Commun.* **16**(1), 1050 (2025). 39865064 10.1038/s41467-025-56433-5 PMC11770113

[15] Jangir, B., Swain, D. & Ghose, S. K. Influence of eddies and tropical cyclone heat potential on intensity changes of tropical cyclones in the North Indian Ocean. *Adv. Space Res.* **68**(2), 773–786 (2021).

[16] Chakraborty, S., Pattnaik, S., Banerjee, A. & Chakraborty, T. Investigation of the contrasting influence of air–sea interactions on the intensification process of pre-and post‐monsoon tropical cyclones over the Bay of Bengal in a coupled model framework. *Q J. R. Meteorol. Soc***e4957**(2025).

[17] Jarugula, S. L. & McPhaden, M. J. Ocean mixed layer response to two post-monsoon cyclones in the Bay of Bengal in 2018. *JGR: Oceans* **127**(9), e2022JC018874 (2022).

[18] Busireddy, N. K. R., Ankur, K., Osuri, K. K., Sivareddy, S. & Niyogi, D. The response of ocean parameters to tropical cyclones in the Bay of Bengal. *Q. J. R Meteorol.* **145**(724), 3320–3332 (2019).

[19] Goswami, B. N., Rao, S. A., Sengupta, D. & Chakravorty, S. Monsoons to mixing in the Bay of Bengal: Multiscale air-sea interactions and monsoon predictability. *Oceanogr* **29**(2), 18–27 (2016).

[20] Mahadevan, A. et al. Freshwater in the Bay of Bengal: Its fate and role in air-sea heat exchange. *Oceanogr* **29**(2), 72–81 (2016).

[21] Roxy, M. K., Ritika, K., Terray, P. & Masson, S. The curious case of Indian Ocean warming. *J. Clim.* **27**(22), 8501–8509 (2014).

[22] Das, B. K. & Pal, A. Spatiotemporal distribution of the upper ocean temperature and salinity in the Bay of Bengal during 1980–2022 and its implications on the mixed layer dynamics. *EFM* **25**(2), 18 (2025).

[23] Nath, S., Kotal, S. D. & Kundu, P. K. Decadal variation of ocean heat content and tropical cyclone activity over the Bay of Bengal. *J. Earth Syst. Sci.* **125**(1), 65–74 (2016).

[24] Wijesekera, H. W. et al. Intraseasonal variability of upper-ocean heat fluxes in the central Bay of Bengal. *J. Phys. Oceanogr.* **52**(2), 261–288 (2022).

[25] Wu, S. N., Soden, B. J. & Alaka, G. J. Jr The influence of radiation on the prediction of tropical cyclone intensification in a forecast model. *Geophys. Res. Lett*. **50**(2), e2022GL099442 (2023).

[26] Maneesha, K., Sadhuram, Y. & Prasad, K. V. S. R. Role of upper ocean parameters in the genesis, intensification and tracks of cyclones over the Bay of Bengal. *J. Oper. Oceanogr.* **8**, 133–146 (2015).

[27] Jana, S., Gangopadhyay, A. & Chakraborty, A. Impact of seasonal river input on the Bay of Bengal simulation. *Cont. Shelf Res.* **104**, 45–62 (2015).

[28] Kumari, A., Kumar, S. P. & Chakraborty, A. Seasonal and interannual variability in the barrier layer of the Bay of Bengal. *JGR: Oceans*. **123**(2), 1001–1015 (2018).

[29] Vinayachandran, P. N., Murty, V. S. N. & Ramesh Babu, V. Observations of barrier layer formation in the Bay of Bengal during summer monsoon. *JGR: Oceans*. **107**(C12), SRF–19 (2002).

[30] Albert, J., Krishnan, A., Bhaskaran, P. K. & Singh, K. S. Role and influence of key atmospheric parameters in large-scale environmental flow associated with tropical cyclogenesis and ENSO in the North Indian Ocean basin. *Clim. Dyn.* **58**(1), 17–34 (2022).

[31] Naskar, P. R., Mohapatra, M. & Singh, G. P. Variations in air-sea heat fluxes during lifetime of intense tropical cyclones over the Bay of Bengal. *Meteorol. Atmos. Phys.* **136**(4), 29 (2024).

[32] Li, Z., Yu, W., Li, T., Murty, V. S. N. & Tangang, F. Bimodal character of cyclone climatology in the Bay of Bengal modulated by monsoon seasonal cycle. *J. Clim.* **26**(3), 1033–1046 (2013).

[33] Chavas, D. R., Reed, K. A. & Knaff, J. A. Physical understanding of the tropical cyclone wind-pressure relationship. *Nat. Commun.* **8**(1), 1360 (2017). 29118342 10.1038/s41467-017-01546-9 PMC5678138

[34] Sengupta, D., Goddalehundi, B. R. & Anitha, D. S. Cyclone-induced mixing does not cool SST in the post‐monsoon north Bay of Bengal. *Atmos. Sci. Lett.* **9**(1), 1–6 (2008).

