# Supplementary material for: Long-term seasonal inhomogeneity and recent patterns in air-sea conditions and tropical cyclone activities over the Bay of Bengal
Source: Sci Rep. 2026 May 12;16:21687. doi: 10.1038/s41598-026-52751-w (PMC13358158; doi:10.1038/s41598-026-52751-w)
Supplement: Supplementary file 1 — Supplementary Material 1 [file 41598_2026_52751_MOESM1_ESM.pdf]

Supplementary Information for

Long-term seasonal inhomogeneity and recent patterns in air-sea conditions and tropical cyclone activities over the Bay of Bengal

Bijan Kumar Das[1,*], Moumita Dinda[1], Anushri Pal[1,2]

[1]Department of Mathematics, Midnapore College (Autonomous), Midnapore-721101, India.

[2]Department of Earth and Atmospheric Sciences, National Institute Technology Rourkela, Rourkela-769008, India.

[*]Corresponding author email ID: bijankumar.das@midnaporecollege.ac.in (Bijan Kumar Das)

ORCID: Bijan Kumar Das (0000-0002-5152-4045), Moumita Dinda (0009-0004-4626-7840), Anushri Pal (0009-0007-9698-9982).

Contains:

Texts, Tables S1-S6

Statistical Analysis: The statistical analysis in this study used the monthly data during 1982-2022 only for Sea Surface Temperature (SST) and Subsurface Temperature (SubST) to show the rapid rise in upper ocean temperature of the Bay of Bengal (BoB) as given in Figure 1a of the manuscript. The seasonal (pre- and post-ISM) variations and the recent (during 2001-2022 compared to 1982-2000) patterns in the ocean, atmospheric and cyclone variables are presented in the statistical analysis using yearly data. Although 1982-2000 (earlier period) includes 19 years and 2001-2022 (recent period) has 22 years, the statistical comparison intends to represent the differences in patterns or changing characteristics between these two periods. In the statistical analysis, the number of data points for the yearly data analysis of the periods 1982-2022, 1982-2000 and 2001-2022 are respectively 41, 19 and 22 (except SST and SubST in Table S1 where monthly mean data is considered).

In the analysis, other than SST and SubST, the variables are Ocean Heat Content (OHC), Cyclone Heat Potential (CHP), 26°C isothermal layer depth (D26), Barrier Layer Thickness (BLT), Long Wave Radiation Flux (LWRF), Latent Heat Flux (LHF), Sensible Heat Flux (SHF), Divergence of Thermal Energy Flux (DTEF), Divergence of Moisture Flux (DMF), Tropical Cyclone (TC) frequency, Maximum Sustained Wind Speed (MSWS), Maximum Pressure Drop (MPD) and Cyclone Duration (CD).

A separate country wise and Indian state wise analysis of TC landfall percentages are also shown here.

Table S1: The yearly statistical values of the upper ocean thermodynamic parameters and Tropical Cyclone (TC) frequency over the BoB during 1982-2022.

1982-2022						
	**Mean**	**Median**	**Standard deviation**	**Range**	**Slope/year (p-value)**	**Increased/ decreased**
**SST (°C)**	28.84	28.86	0.743	3.912	0.016 (6.79×10^{-9})	0.66
**SubST (°C)**	27.08	27.07	0.42	2.61	0.013 (5.97×10^{-18})	0.54
**OHC ($\times10^{20}$ J)**	428	429	3.76	17.28	0.22 5.73×10^{-14}	9.0
**CHP ($\times10^{19}$ J)**	241.4	241.6	26.14	109.4	1.69 (2.1×10^{-16})	67.0
**TC frequency (per year)**	2.829	3	1.302	5	-0.012 (0.498)	-0.48

Table S2: The seasonal (pre- and post-ISM) statistical values of the upper ocean thermodynamic parameters in the BoB during 1982-2022, 1982-2000 and 2001-2022.

Ocean parameters												
**1982-2022**												
	**Pre-ISM**						**Post-ISM**					
	**Mean**	**Median**	**Standard deviation**	**Range**	**Slope/year (p-value)**	**Increased/ decreased**	**Mean**	**Median**	**Standard deviation**	**Range**	**Slope/year (p-value)**	**Increased/ decreased**
**SST (°C)**	29.57	29.53	0.31	1.34	0.017 (3.25×10^{-6})	0.7	28.67	28.71	0.3	1.24	0.016 (5.52×10^{-6})	0.7
**SubST (°C)**	27.41	27.38	0.26	1.41	0.012 (9.66×10^{-5})	0.5	26.98	26.98	0.29	1.43	0.014 (6.04×10^{-5})	0.6
**D26 (m)**	71.65	70.77	5.12	25.49	0.2 (0.00183)	8.1	68.06	67.93	4.95	22.6	0.21 (0.00058)	8.5
**BLT (m)**	8.75	8.49	1.27	6	-0.005 (0.782)	-0.19	16.84	16.85	2.18	10.88	0.045 (0.123)	1.7
**OHC ($\times10^{19}$ J)**	2.16	2.15	0.02	0.125	0.001 (6.02×10^{-14})	0.04	2.13	2.13	0.03	0.14	0.0012 (5.5×10^{-14})	0.05
**CHP ($\times10^{18}$ J)**	1.35	1.35	0.14	0.59	0.0085 (4.50×10^{-16})	0.34	1.16	1.16	0.16	0.64	0.0093 (1.18×10^{-16})	0.37
**1982-2000**												
	**Pre-ISM**						**Post-ISM**					
	**Mean**	**Median**	**Standard deviation**	**Range**	**Slope/year (p-value)**	**Increased/ decreased**	**Mean**	**Median**	**Standard deviation**	**Range**	**Slope/year (p-value)**	**Increased/ decreased**
**SST (°C)**	29.37	29.32	0.24	0.83	0.0026 (0.808)	0.1	28.47	28.38	0.26	0.94	~0 (0.991)	0
**SubST (°C)**	27.25	27.27	0.2	0.9	-0.005 (0.594)	-0.1	26.81	28.76	0.25	1.14	~0 (0.998)	0
**D26 (m)**	69.43	69.51	4.07	17.29	~0 (0.998)	0	65.63	65.51	4.56	21.03	0.026 (0.896)	0.5
**BLT (m)**	8.77	8.87	1.26	5.07	0.074 (0.166)	1.34	16.62	16.04	2.7	10.88	0.24 (0.026)	4.4
**OHC ($\times10^{19}$ J)**	2.15	2.14	0.018	0.088	-0.0002 (5.76×10^{-7})	-0.01	2.11	2.11	0.02	0.11	0 (5.68×10^{-7})	0
**CHP ($\times10^{18}$ J)**	1.25	1.27	0.089	0.32	0.0044 (3.48×10^{-7})	0.08	1.04	1.02	0.12	0.43	0.003 (4.88×10^{-7})	0.06
**2001-2022**												
	**Pre-ISM**						**Post-ISM**					
	**Mean**	**Median**	**Standard deviation**	**Range**	**Slope/year (p-value)**	**Increased/ decreased**	**Mean**	**Median**	**Standard deviation**	**Range**	**Slope/year (p-vale)**	**Increased/ decreased**
**SST (°C)**	29.73	29.72	0.26	1.0	0.23 (0.00683)	0.5	28.84	28.84	0.2	0.88	0.016 (0.018)	0.3
**SubST (°C)**	27.54	27.49	0.24	0.86	0.015 (0.0516)	0.3	27.12	27.15	0.24	0.99	0.017 (0.0336)	0.4
**D26 (m)**	73.56	72.48	5.23	17.69	0.33 (0.0548)	7.1	70.16	69.81	4.33	15.47	0.29 (0.043)	6.1
**BLT (m)**	8.74	8.23	1.32	5.22	-0.071 (0.109)	-1.5	17.04	17.01	1.65	7.45	0.032 (0.572)	0.7
**OHC ($\times10^{19}$ J)**	2.17	2.16	0.02	0.075	0.001 (5.74×10^{-8})	0.02	2.14	2.14	0.02	0.087	0.0015 (5.52×10^{-8})	0.03
**CHP ($\times10^{18}$ J)**	1.44	1.42	0.11	0.4	0.0089 (1.66×10^{-8})	0.18	1.25	1.23	0.12	0.48	0.01 (1.04×10^{-8})	0.22

Table S3: The seasonal (pre- and post-ISM) statistical values of the atmospheric parameters over the BoB during 1982-2022, 1982-2000 and 2001-2022.

Atmospheric parameters												
**1982-2022**												
	**Pre-ISM**						**Post-ISM**					
	**Mean**	**Median**	**Standard deviation**	**Range**	**Slope/year (p-value)**	**Increased/ decreased**	**Mean**	**Median**	**Standard deviation**	**Range**	**Slope/year (p-value)**	**Increased/ decreased**
**LWRF (Watt/m^2)**	57.89	57.5	2.34	12.11	-0.063 (0.0382)	-2.6	54.31	54.34	2.01	7.03	-0.1 (6.18×10^{-5})	-3.9
**LHF (Watt/m^2)**	114.3	114.5	5.21	22.55	0.14 (0.0439)	5	126.5	126.4	5.03	20.52	0.02 (0.768)	1
**SHF (Watt/m^2)**	10.8	10.82	0.5	1.99	-0.015 (0.0177)	-0.6	11.69	11.68	0.59	2.4	-0.027 (0.000244)	-1
**DTEF (Watt/m^3)**	0.29	0.254	0.17	0.59	-0.0017 (0.456)	-0.067	0.204	0.22	0.15	0.642	-0.0025 (0.2)	-0.1
**DMF ($\times10^{-9}$ s^{-1})**	1.96	1.45	4.61	18.2	-0.018 (0.776)	-0.7	4.96	4.43	4.74	21.78	-0.078 (0.215)	-3.13
**1982-2000**												
	**Pre-ISM**						**Post-ISM**					
	**Mean**	**Median**	**Standard deviation**	**Range**	**Slope/year (p-value)**	**Increased/ decreased**	**Mean**	**Median**	**Standard deviation**	**Range**	**Slope/year (p-value)**	**Increased/ decreased**
**LWRF (Watt/m^2)**	58.77	58.84	2.82	11.04	-0.018 (0.887)	-0.3	55.3	55.87	1.67	6.22	-0.063 (0.381)	-1.2
**LHF (Watt/m^2)**	112.2	111.3	5.3	22.55	0.61 (0.00285)	11	126.4	125.6	5.62	20.52	0.38 (0.103)	7
**SHF (Watt/m^2)**	10.88	10.85	0.44	1.54	-0.003 (0.877)	0	11.94	11.94	0.53	2	-0.018 (0.434)	-0.3
**DTEF (Watt/m^3)**	0.317	0.317	0.153	0.479	0.0021 (0.757)	0.067	0.22	0.22	0.14	0.576	-0.0081 (0.184)	-0.15
**DMF ($\times10^{-9}$ s^{-1})**	2.41	2.7	3.98	13.58	0.11 (0.523)	1.99	5.55	4.43	5.01	21.78	-0.275 (0.198)	-4.95
**2001-2022**												
	**Pre-ISM**						**Post-ISM**					
	**Mean**	**Median**	**Standard deviation**	**Range**	**Slope/year (p-value)**	**Increased/ decreased**	**Mean**	**Median**	**Standard deviation**	**Range**	**Slope/year (p-value)**	**Increased/ decreased**
**LWRF (Watt/m^2)**	57.13	57.08	1.52	6.64	-0.014 (0.79)	-0.3	53.45	53.2	1.9	6.53	-0.16 (0.0109)	-3.3
**LHF (Watt/m^2)**	116.1	115.9	4.5	15.33	-0.42 (0.00263)	-8	126.6	126.8	4.6	15.27	-0.16 (0.3)	-3
**SHF (Watt/m^2)**	10.74	10.62	0.54	1.99	-0.064 (4.04e-05)	-1.3	11.48	11.37	0.57	2.06	-0.054 (0.00239)	-1.1
**DTEF (Watt/m^3)**	0.255	0.225	0.18	0.59	0.0025 (0.686)	0.052	0.19	0.2	0.15	0.53	-0.0053 (0.314)	-0.112
**DMF ($\times10^{-9}$ s^{-1})**	1.57	0.77	5.14	17.36	0.0012 (0.948)	0.25	4.46	4.26	4.56	14.93	-0.072 (0.651)	-1.51

Table S4: The seasonal (pre- and post-ISM) statistical values of the cyclone parameters over the BoB during 1982-2022, 1982-2000 and 2001-2022.

Cyclone parameters												
**1982-2022**												
	**Pre-ISM**						**Post-ISM**					
	**Mean**	**Median**	**Standard deviation**	**Range**	**Slope/year (p-value)**	**Increased/ decreased**	**Mean**	**Median**	**Standard deviation**	**Range**	**Slope/year (p-value)**	**Increased/ decreased**
**Frequency (per year)**	0.659	1	0.617	2	0.0098 (0.235)	0.391	1.829	2	1.09	4	-0.018 (0.227)	-0.7
**MSWS (Km/h)**	87.36	83.34	86.26	240.8	0.405 (0.727)	16.2	129	120.4	59.9	259.3	-0.78 (0.33)	-32
**MPD (hPa)**	21.88	10	27.1	84	-0.073 (0.841)	-2.9	30.5	26	23.4	98	-0.371 (0.234)	-14.8
**CD (hours)**	59.37	54	60.81	225	1.24 (0.124)	49.5	135.9	117	99.7	426	0.667 (0.618)	26
**1982-2000**												
	**Pre-ISM**						**Post-ISM**					
	**Mean**	**Median**	**Standard deviation**	**Range**	**Slope/year (p-value)**	**Increased/ decreased**	**Mean**	**Median**	**Standard deviation**	**Range**	**Slope/year (p-value)**	**Increased/ decreased**
**Frequency (per year)**	0.579	1	0.607	2	0.014 (0.595)	0.252	2.105	2	1.1	3	-0.016 (0.742)	-0.29
**MSWS (Km/h)**	87.14	64.82	96.67	235.2	-0.12 (0.977)	-2.1	148.2	142.6	56.8	194.5	2.2 (0.37)	40
**MPD (hPa)**	24.16	10	31.19	80	-0.449 (0.742)	-8.1	38.45	40	25.1	90	0.916 (0.398)	16.5
**CD (hours)**	44.74	33	49.19	132	-0.137 (0.949)	-2.5	143.8	138	88.5	288	4.73 (0.211)	85
**2001-2022**												
	**Pre-ISM**						**Post-ISM**					
	**Mean**	**Median**	**Standard deviation**	**Range**	**Slope/year (p-value)**	**Increased/ decreased**	**Mean**	**Median**	**Standard deviation**	**Range**	**Slope/year (p-value)**	**Increased/ decreased**
**Frequency (per year)**	0.727	1	0.631	2	0.019 (0.378)	0.403	1.591	1	1.05	4	0.018 (0.633)	0.36
**MSWS (Km/h)**	87.55	92.6	78.5	240.8	2.6 (0.336)	54.8	112.4	101.9	58.8	213	1.97 (0.33)	41.3
**MPD (hPa)**	19.91	13	23.59	84	0.819 (0.313)	17.2	23.64	14.5	20	66	0.501 (0.468)	10.5
**CD (hours)**	72	87	67.88	225	1.68 (0.475)	35.2	129.1	111	110	426	4.74 (0.208)	99.6

Table S5: Country wise cyclone landfall numbers and percentages during pre- and post-ISM of 1998-2022, 1982-2000 and 2001-2022. SL = Sri Lanka, IN = India, BD = Bangladesh, MM = Myanmar.

**Country wise cyclone landfall percentage (with TC number)**										
**Period**	**Pre-ISM**					**Post-ISM**				
	**Total**	**SL**	**IN**	**BD**	**MN**	**Total**	**SL**	**IN**	**BD**	**MN**
**1982-2022**	100 (27)	0	29.63 (8)	44.44 (12)	25.93 (7)	100 (75)	2.67 (2)	77.33 (58)	14.67 (11)	5.33 (4)
**1982-2000**	100 (11)	0	18.18 (2)	63.64 (7)	18.18 (2)	100 (40)	2.5 (1)	70 (28)	20 (8)	7.5 (3)
**2001-2022**	100 (16)	0	37.5 (6)	31.25 (5)	31.25 (5)	100 (35)	2.86 (1)	85.71 (30)	8.57 (3)	2.86 (1)

Table S6: Indian states wise cyclone landfall numbers and percentages during pre- and post-ISM of 1998-2022, 1982-2000 and 2001-2022. TN = Tamil Nadu, PY = Pondicherry, AP = Andhra Pradesh; OD = Odissa, WB = West Bengal.

**State wise cyclone landfall percentage in India (with TC number)**											
**Period**	**Pre-ISM**						**Post-ISM**				
	**Total**	**TN**	**PY**	**AP**	**OD**	**WB**	**Total**	**TN**	**PY**	**AP**	**OD**	**WB**
**1982-2022**	100 (8)	0	0	37.5 (3)	37.5 (3)	25 (2)	100 (58)	34.48 (20)	1.72 (1)	37.93 (22)	15.52 (9)	10.34 (6)
**1982-2000**	100 (2)	0	0	50 (1)	50 (1)	0	100 (28)	17.86 (5)	3.57 (1)	42.86 (12)	21.43 (6)	14.29 (4)
**2001-2022**	100 (6)	0	0	33.33 (2)	33.33 (2)	33.33 (2)	100 (30)	50 (15)	0	33.33 (10)	10 (3)	6.67 (2)
